# Eukaryotic transporters for hydroxyderivatives of benzoic acid

**DOI:** 10.1038/s41598-017-09408-6

**Published:** 2017-08-21

**Authors:** Andrea Cillingová, Igor Zeman, Renáta Tóth, Martina Neboháčová, Ivana Dunčková, Mária Hölcová, Michaela Jakúbková, Gabriela Gérecová, Leszek P. Pryszcz, Ľubomír Tomáška, Toni Gabaldón, Attila Gácser, Jozef Nosek

**Affiliations:** 1 Departments of Biochemistry and Genetics, Comenius University in Bratislava, Faculty of Natural Sciences, Ilkovičova 6, 842 15 Bratislava, Slovak Republic; 2 Department of Microbiology, University of Szeged, Szeged, Közép fasor 52, H-6726 Szeged, Hungary; 3 Bioinformatics and Genomics Programme, Centre for Genomic Regulation, Doctor Aiguader 88, 08003 Barcelona, Spain; 4 Departament de Ciències Experimentals I de la Salut, Universitat Pompeu Fabra, 08003 Barcelona, Spain; 5 Institució Catalana de Recerca i Estudis Avançats, Pg. Lluís Companys 23, 08010 Barcelona, Spain; 6 Department of Biochemistry and Cell Biology, Present Address: Max F. Perutz Laboratories, University of Vienna, Dr. Bohr Gasse 9, 1030 Vienna, Austria; 7 Present Address: International Institute of Molecular and Cell Biology in Warsaw, 4 Trojdena Street, 02-109 Warsaw, Poland

## Abstract

Several yeast species catabolize hydroxyderivatives of benzoic acid. However, the nature of carriers responsible for transport of these compounds across the plasma membrane is currently unknown. In this study, we analyzed a family of genes coding for permeases belonging to the major facilitator superfamily (MFS) in the pathogenic yeast *Candida parapsilosis*. Our results revealed that these transporters are functionally equivalent to bacterial aromatic acid: H^+^ symporters (AAHS) such as GenK, MhbT and PcaK. We demonstrate that the genes *HBT1* and *HBT2* encoding putative transporters are highly upregulated in *C. parapsilosis* cells assimilating hydroxybenzoate substrates and the corresponding proteins reside in the plasma membrane. Phenotypic analyses of knockout mutants and hydroxybenzoate uptake assays provide compelling evidence that the permeases Hbt1 and Hbt2 transport the substrates that are metabolized via the gentisate (3-hydroxybenzoate, gentisate) and 3-oxoadipate pathway (4-hydroxybenzoate, 2,4-dihydroxybenzoate and protocatechuate), respectively. Our data support the hypothesis that the carriers belong to the AAHS family of MFS transporters. Phylogenetic analyses revealed that the orthologs of Hbt permeases are widespread in the subphylum Pezizomycotina, but have a sparse distribution among Saccharomycotina lineages. Moreover, these analyses shed additional light on the evolution of biochemical pathways involved in the catabolic degradation of hydroxyaromatic compounds.

## Introduction

The major facilitator superfamily (MFS) proteins represent the largest group of secondary carriers involved in various transport processes including uniport, solute: cation symport and solute: H^+^ or solute: solute antiport. These permeases are usually 400–600 amino acids long, form either 12 or 14 transmembrane α-helices (TMH) and exhibit specificity for a wide range of substrates including sugars, amino acids, nucleosides, lipids, metabolic intermediates, ions and drugs. They are classified into about 100 families based on their sequence similarity, substrate specificity and mechanism of action (reviewed in refs 1–7). Bacterial MFS transporters mediating the uptake of aromatic acids belong to several families including ACS (anion: cation symporter; *e.g*. OphD, OphP, Pht1)^8–10^, AAHS (aromatic acid: H^+^ symporter; *e.g*. BenK, GenK, MhbT, PcaK)^11–14^ and MHS (metabolite: H^+^ symporter; *e.g*. MopB, PcaT, ShiA)^15–17^. Moreover, carriers from other superfamilies such as ABC (ATP-binding cassette; *e.g*. OphFGH)^10^, APC (amino acid-polyamine-organocation; *e.g*. BenE)^18^ and OMPP (outer membrane pore-forming protein; *e.g*. BenP)^19^ also transport aromatic substrates.

Pioneering studies^20, 21^ on the basidiomycete *Trichosporon cutaneum* uncovered an inducible energy-dependent uptake system for phenol and demonstrated that phenolate anions are co-transported with protons in stoichiometry 1:1. The symport with H^+^ ion was also shown for vanillate in another basidiomycete *Fomitopsis palustris* ^22^. However, the genes encoding these transporters have not yet been identified. In human cells, sodium-coupled monocarboxylate transporter for short-chain fatty acids (SMCT1, SLC5A8) is involved in the uptake of nicotinate and various aromatic monocarboxylates including benzoate and salicylate^23^.

In this study, we analyzed the genes from the pathogenic yeast *Candida parapsilosis* that encode MFS permeases for hydroxyderivatives of benzoic acid. This yeast utilizes various hydroxyaromatic substrates via the gentisate (*i.e*. 3-hydroxybenzoate, 2,5-dihydroxybenzoate (gentisate)) and 3-oxoadipate (*i.e*. hydroquinone, resorcinol, 4-hydroxybenzoate, 2,4-dihydroxybenzoate (β-resorcylate) and 3,4-dihydroxybenzoate (protocatechuate)) pathway^24–26^. The range of catabolized substrates differs among closely related species that are classified into the CTG clade of Saccharomycotina. For example, *Candida orthopsilosis* assimilates hydroxybenzenes and hydroxybenzoates via the 3-oxoadipate pathway, but lacks the gentisate pathway. On the other hand, *Candida albicans, Candida dubliniensis* and *Candida tropicalis* catabolize mono- and dihydroxybenzenes, but not hydroxybenzoates^26^. This metabolic diversity indicates that *C. parapsilosis* possesses functional transporter(s) for hydroxybenzoates and the species like *C. albicans* may lack such carriers. As the genomes of several CTG clade species were sequenced and annotated^27–34^, hydroxybenzoate transporter genes could be identified by means of comparative and functional genomics.

The *C. parapsilosis* genome encodes 138 predicted MFS proteins (Pfam clan CL0015). Although they have not been functionally characterized, computational analyses indicated role(s) for many of them based on their sequence homology to transporters with known functions^35–37^. However, none of them has a predicted role in the uptake of hydroxybenzoates. Moreover, BlastP searches with queries derived from bacterial hydroxybenzoate permeases (*i.e*. MhbT for 3-hydroxybenzoate^13^, PcaK for 4-hydroxybenzoate and protocatechuate^12^ and GenK for gentisate^14^) did not reveal any significant hit. We therefore reasoned that, similarly to the enzymes catalyzing reactions of the gentisate and 3-oxoadipate pathways^26, 38, 39^, the hydroxybenzoate transporters could be upregulated in *C. parapsilosis* cells grown in media containing a hydroxybenzoate as a sole carbon source. Indeed, an RNA-seq experiment revealed two candidate genes CPAR2_704330 and CPAR2_204840 coding for predicted MFS transporters that are highly induced in the cells assimilating 3-hydroxybenzoate and 4-hydroxybenzoate, respectively. Although corresponding proteins were previously classified into drug: H^+^ antiporter family 1 (DHA1)^37^, results of the functional analysis clearly demonstrate that both permeases are involved in the uptake of hydroxybenzoates presumably by proton symport mechanism.

## Results and Discussion

### Identification of the genes for hydroxybenzoate transporters

Initially we attempted to search for candidate genes encoding the hydroxybenzoate transporters by blasting the *C. parapsilosis* genome using the amino acid sequences of bacterial transporters for aromatic acids classified into different superfamilies such as ABC, APC, MFS and OMPP (Supplementary Table S1). The searches with all but one bacterial query did not reveal any clear candidate for hydroxybenzoate carriers (E-values were above 10^−25^). In case of the phthalate permease OphD from *Burkholderia cepacia*, the best identified hits were CPAR2_802720 and its three paralogs CPAR2_802710, CPAR2_802700 and CPAR2_802690 (E-values were 2 × 10^−35^ to 2 × 10^−30^). These genes are tandemly arranged on the chromosomal contig005807 and the analysis of deduced amino acid sequences predicted MFS_1 domain (PF07690) and twelve TMHs (except CPAR2_802690, which appears to be truncated at its N-terminus). Similarly to OphD, the *C. parapsilosis* proteins as well as the *C. albicans* CR_01220 W (an ortholog of CPAR2_802720) can be classified into the ACS family of MFS transporters^9, 35^.

Next, we compared the expression of predicted plasma membrane transporter genes in cells assimilating 3-hydroxybenzoate, 4-hydroxybenzoate or glucose by RNA-seq analysis. Our results showed that the transporter gene with the highest level of expression in the control cells grown in synthetic medium containing glucose (SD) was CPAR2_212860 encoding an MFS protein with predicted sugar transporter domain (PF00083/Sugar_tr). Its ortholog in *C. albicans* is *HGT7* (C2_01000 W) and codes for a glucose transporter^40, 41^. We assumed that CPAR2_212860 may have similar function in *C. parapsilosis*. In contrast to SD medium, the most expressed genes in synthetic media containing a hydroxybenzoate are CPAR2_704330 (S3OH) and CPAR2_204840 (S4OH) that code for uncharacterized members of the MFS. These genes have no orthologs in *C. albicans*, which metabolizes hydroxybenzenes, but does not assimilate hydroxybenzoates, further supporting the idea that they are associated with the hydroxybenzoate metabolism. The expression of both genes was nearly undetectable on glucose, but they were highly upregulated in media containing a hydroxybenzoate (Supplementary Table S2). CPAR2_704330 was induced more than 1,402-fold on S3OH and CPAR2_204840 more than 1,247-fold on S4OH. Such strong induction was observed only on one hydroxybenzoate indicating that corresponding transporters could be specific for the substrate present in the cultivation medium.

Of the four OphD homologs identified by the BlastP searches, the highest level of expression has CPAR2_802720 on S4OH medium, where it exhibits 1,378-fold induction. In spite of such high induction, the overall transcript level of CPAR2_802720 was more than 14 times lower than observed for CPAR2_204840 and its paralogs (*i.e*. CPAR2_802710, CPAR2_802700 and CPAR2_802690) were weakly expressed on all three media (Supplementary Table S2).

BlastP searches using CPAR2_704330 and CPAR2_204840 as queries identified additional two uncharacterized MFS proteins, CPAR2_100470 and CPAR2_100460. These hits had E-values below 10^−80^, while all remaining hits had E-values above 10^−25^. The amino acid sequences of CPAR2_704330, CPAR2_204840, CPAR2_100470 and CPAR2_100460 display extensive sequence similarity (Supplementary Fig. S1), possess a typical MFS_1 domain and twelve TMHs (Supplementary Fig. S2). All four proteins belong to the Pfam clan CL0015 (Supplementary Table S2). Previously, they were classified into the drug: H^+^ antiporter 1 (DHA1) family (2.A.1.2)^37^. However, our phylogenetic analysis indicates that the sequences of all four *C. parapsilosis* proteins cluster with typical AAHS permeases (Supplementary Fig. S3). Therefore, we propose that these proteins are eukaryotic members of the AAHS family (2.A.1.15). Their sequences exhibit less than 20% overall identity with the bacterial AAHS carriers such as BenK, GenK, PcaK and MhbT and lack a typical ‘DGXD’ motif present in the TMH 1. Nonetheless, a more detailed sequence comparison revealed several similarities with the bacterial transporters. The hydrophilic regions between the TMHs 2-3 and 8-9 contain conserved motifs ‘VPXMX(R/A)YG(K/R)(R/K)’ and ‘G(Y/P)(M/L)SDX(L/W)(V/M)X(W/R)’, respectively (Supplementary Fig. S4). These motifs resemble to the consensus ‘GXXXD(R/K)XGR(R/K)’, which in bacterial MFS permeases has a role in the substrate transport^42^. Importantly, the aspartate residue in the motif present within the loop between TMHs 8 and 9, which is conserved in the entire AAHS family, occurs also in the *C. parapsilosis* transporters. In addition, charged residues R124, E144, R386 and R398 present in the sequence of *Pseudomonas putida* PcaK^43^ were found also in the *C. parapsilosis* proteins (*i.e*. the arginines occur in the positions corresponding to R124 and R398; a glutamic acid is in E144 (except for CPAR2_704330, which has an aspartic acid in this position); and a lysine replaces R386). Moreover, the amino acid sequence alignment revealed several additional residues (*i.e*. G92, G165, P287, G310, G368 according to the PcaK numbering) that are shared by bacterial and *C. parapsilosis* proteins (Supplementary Fig. S1). Importantly, some of the amino acid residues conserved between the bacterial AAHS and *C. parapsilosis* proteins were shown to be essential for activity of the AAHS transporters (Supplementary Table S3). Based on the sequence analysis and the experimental results shown below we named these genes as *HBT1* (for hydroxybenzoate transporter 1; CPAR2_704330), *HBT2* (CPAR2_204840), *HBT3* (CPAR2_100470) and *HBT4* (CPAR2_100460).

The *HBT1* gene is located between the genes *MNX2* (CPAR2_704320) and *GDX1* (CPAR2_704340), within the metabolic gene cluster coding for the gentisate pathway enzymes^26^ and the phylogenetic analysis showed that it co-evolved with other genes present in this cluster^39^ further supporting its association with the gentisate pathway.

### Quantitative RT-PCR analysis of the expression of *HBT* genes

To confirm the data obtained by the RNA-seq experiment and to further investigate the expression of *HBT1*-*HBT4* genes, we analyzed the levels of corresponding transcripts by qPCR in cells cultivated in synthetic media containing a hydroxyaromatic compound (*i.e*. 3-hydroxybenzoate, 4-hydroxybenzoate, 2,4-dihydroxybenzoate, gentisate, protocatechuate, hydroquinone and resorcinol), a respiratory substrate (glycerol and ethanol) or glucose as a sole carbon source. In these assays, we used the expression of *MNX1* (4-hydroxybenzoate 1-hydroxylase) and *MNX2* (3-hydroxybenzoate 6-hydroxylase) as markers showing the activation of the 3-oxoadipate and gentisate pathway, respectively. *MNX1* is strongly induced on the substrates of the 3-oxoadipate pathway (1,243-fold on 4-hydroxybenzoate, 1,101-fold on protocatechuate, 886-fold on 2,4-dihydroxybenzoate, 113-fold on resorcinol and 66-fold on hydroquinone), but also on gentisate (259-fold) and to a lesser extent on 3-hydroxybenzoate (15.6-fold), which are catabolized via the gentisate pathway. In contrast, *MNX2* is highly upregulated on 3-hydroxybenzoate (884-fold) and gentisate (240-fold) but it exhibits lower induction on hydroquinone (75-fold), 4-hydroxybenzoate (30-fold), protocatechuate (9.8-fold) and resorcinol (3.2-fold).

**Figure 1:**
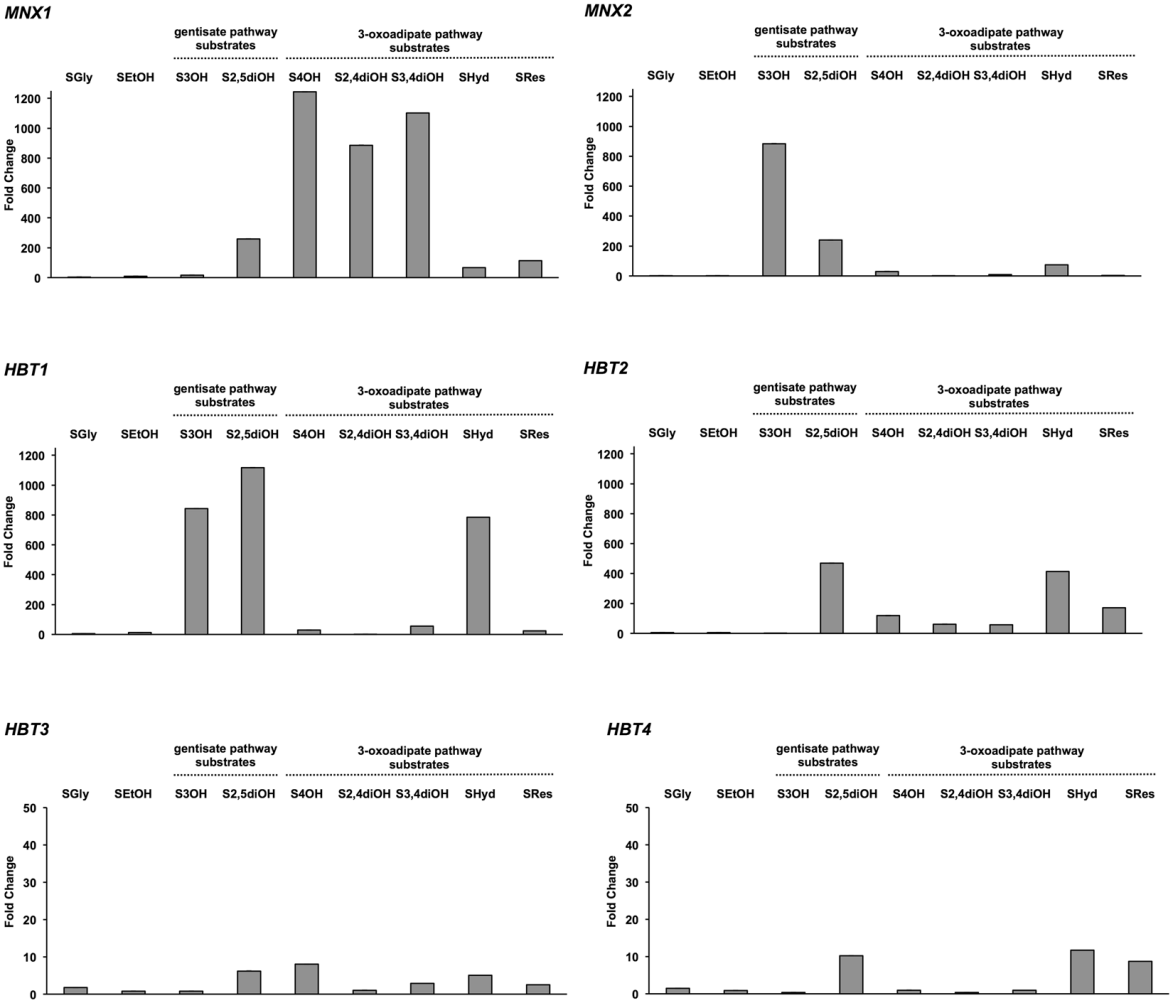
Relative mRNA expression of *C. parapsilosis* genes coding for hydroxybenzoate transporters (*HBT1-4*) and monooxygenases (*MNX1-2*). Bar graph showing the fold change of the *HBT1-4, MNX1* and *MNX2* mRNA levels in cells grown on depicted substrates relative to the mRNA levels in cells assimilating glucose (SD). The assays were performed in at least three independent experiments with two parallel replicates in each case and the values of mean and SEM/SD are shown in Supplementary Table S4.

The expression of *HBT1* is strongly induced on both gentisate pathway substrates (*i.e*. 1,117-fold on gentisate and 843-fold on 3-hydroxybenzoate), but also on hydroquinone (785-fold), which enters the 3-oxoadipate pathway. However, this gene exhibits only modest induction in media with the remaining hydroxyaromatic substrates. *HBT2* is induced on all substrates degraded via the 3-oxoadipate pathway (*i.e*. 414-fold on hydroquinone, 171-fold on resorcinol, 118-fold on 4-hydroxybenzoate, 61-fold on 2,4-dihydroxybenzoate and 57-fold on protocatechuate) as well as on gentisate (489-fold). The induction of both *HBT3* and *HBT4* is noticeably lower when compared to *HBT1* or *HBT2*. We observed a slight induction of *HBT3* on 4-hydroxybenzoate (8.1-fold), gentisate (6.2-fold), hydroquinone (5.1-fold), protocatechuate (2.9-fold) and resorcinol (2.5-fold). *HBT4* was induced on hydroquinone (11.7-fold), gentisate (10.2-fold) and resorcinol (8.7-fold) (Fig. 1, Supplementary Table S4). These results demonstrate that all four *HBT* genes are upregulated in a substrate-dependent manner in cells assimilating a hydroxyaromatic compound.

### Phenotypic analysis of *Δhbt* mutants

The expression profiles of *HBT* genes indicated that corresponding protein products could be involved in the uptake of hydroxyaromatic compounds. To test this idea, we constructed a set of homozygous knockout strains each lacking a single *HBT* gene. In standardized phenotypic tests (Supplementary Table S5) we observed that the growth of *Δhbt1/Δhbt1*, *Δhbt2/Δhbt2* and *Δhbt3/Δhbt3* strains in complex (YPD) as well as synthetic media (SD_1%_, SD_1%_ + FBS, YCB + BSA), the colony morphology, the formation of pseudohyphae, the sensitivity to oxidative stress (H_2_O_2_), detergents (SDS), the inhibitors of cell wall biosynthesis (calcofluor white, congo red, caffeine), hygromycin B and antifungal drugs (caspofungin, fluconazole) remain unchanged when compared to the wild type strain. The phenotype of the *Δhbt4/Δhbt4* mutant was similar, except its slower growth in synthetic media at 20 °C, formation of smaller colonies on SD_1%_ plates, resistance to caffeine and altered sensitivity to both antifungals (Supplementary Table S5, Supplementary Fig. S5).

**Figure 2:**
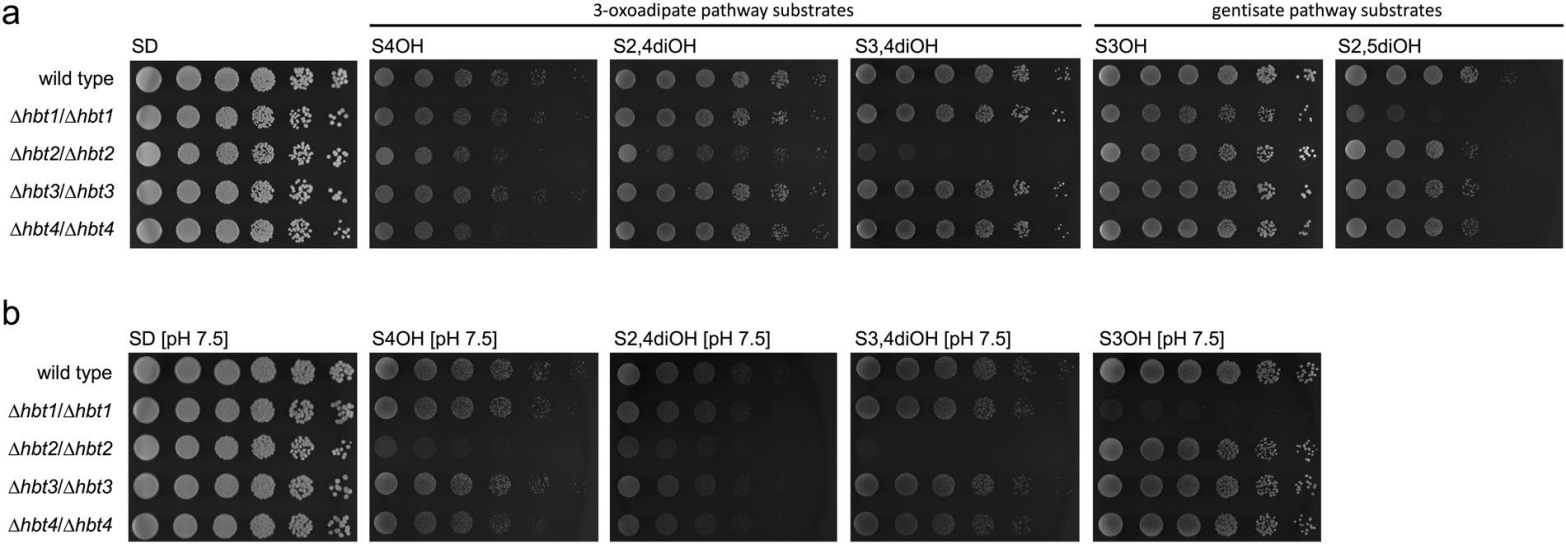
Utilization of hydroxybenzoates by *C. parapsilosis* mutants. The wild type strain CLIB214 and the *hbt* mutants were grown overnight in liquid YPD medium at 28 °C, washed with water, diluted to a concentration 6 × 10^6^ cells/ml. Serial fivefold dilutions were then spotted on the synthetic media containing either glucose or a hydroxybenzoate substrate as a sole carbon source. The plates were incubated for 4 days at 28 °C. The yeast strains were cultivated in unbuffered media (**a**) as well as in the media with pH adjusted to 7.5 using 100 mM Tris-HCl (**b**).

Next, we tested the ability of mutant strains to grow in synthetic media containing a hydroxybenzoate as a sole carbon source (Fig. 2a,b). We observed impaired growth of the mutants *Δhbt1*/*Δhbt1* and *Δhbt2*/*Δhbt2* in media with substrates assimilated via the gentisate and 3-oxoadipate pathway, respectively. The phenotypes were more pronounced in media buffered to pH 7.5 (Fig. 2b) possibly reflecting the fact that pKa values of all hydroxybenzoates are lower than 5. This may cause that in non-buffered media (at pH below 3.5) the hydroxyaromatic substrates are at least partially present in their undissociated form, which may enter the cells by a simple diffusion. In contrast to strains *Δhbt1/Δhbt1* and *Δhbt2/Δhbt2*, the mutants *Δhbt3/Δhbt3* and *Δhbt4/Δhbt4* do not exhibit growth defect in these media. This may suggest that Hbt3 and Hbt4 are not involved in hydroxybenzoate uptake or their affinity to these compounds substantially differ from that of Hbt1 and Hbt2 (*e.g*. Hbt1 and Hbt2 may represent high-affinity and Hbt3 and Hbt4 low-affinity transporters). In the latter case, Hbt1 and/or Hbt2 would compensate the growth defects resulting from the absence of Hbt3 and Hbt4 proteins.

**Figure 3:**
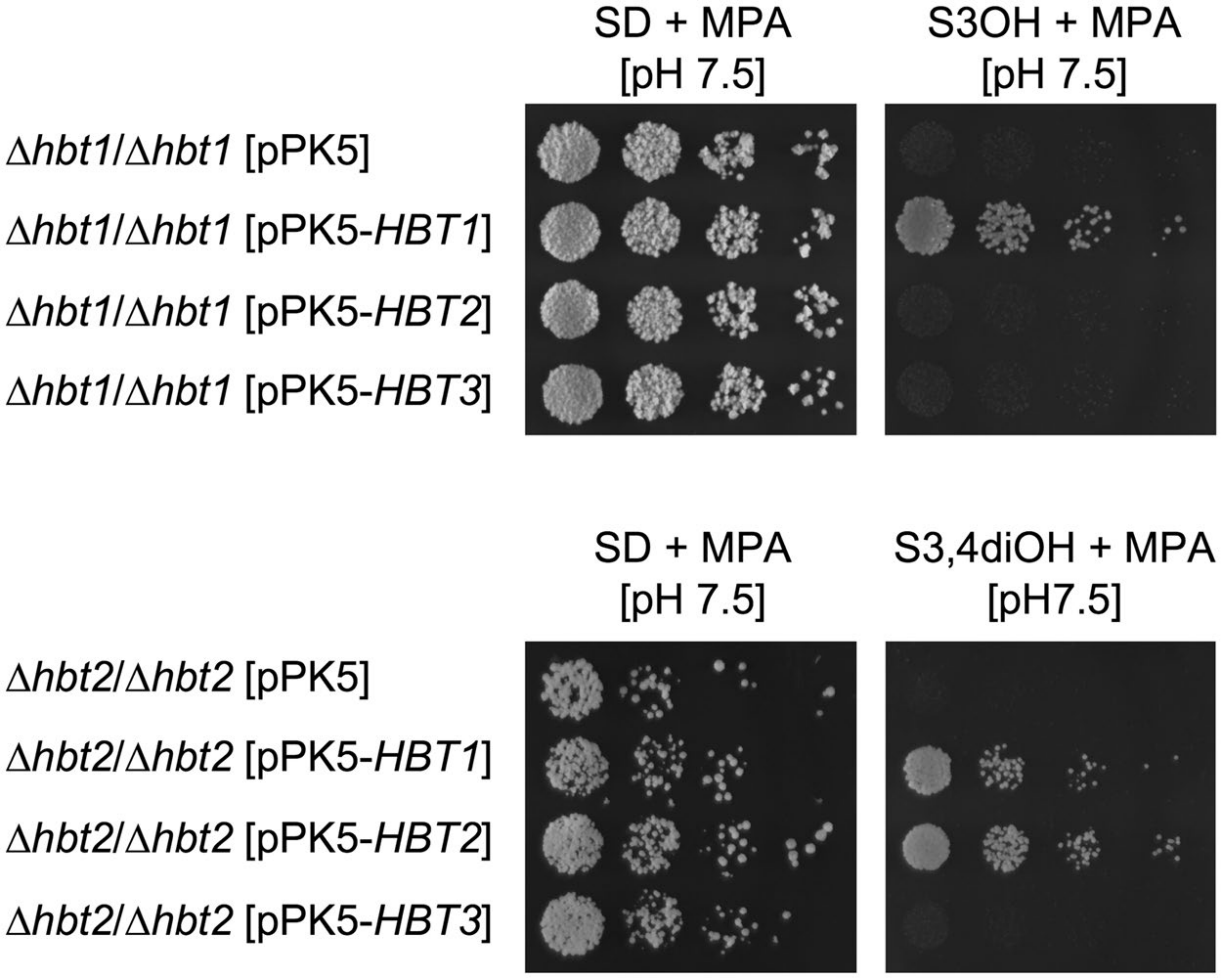
Functional complementation of the *Δhbt1* and *Δhbt2* mutations. The mutants *Δhbt1/Δhbt1* and *Δhbt2/Δhbt2* were transformed with the pPK5-derived plasmid constructs carrying either *HBT1*, *HBT2* or *HBT3* gene. The transformants were cultivated overnight in SD + MPA medium at 28 °C, washed with water, diluted to concentration 5 × 10^5^ cells/ml and plated in fivefold dilutions onto indicated synthetic media buffered with 100 mM Tris-HCl to pH 7.5. The plates were incubated for 7 days at 28 °C. Transformants with the vector pPK5 were used as a control.

To verify that the phenotypes of *Δhbt1/Δhbt1* and *Δhbt2/Δhbt2* strains are caused by deletions of the transporter gene and to localize corresponding proteins within the *C. parapsilosis* cells, we transformed both mutants using the plasmids expressing the Hbt proteins tagged with the yEGFP3 at their C-termini. This experiment confirmed that pPK5-*HBT1* and pPK5-*HBT2* complement corresponding mutations. In addition, we observed that pPK5-*HBT1* suppresses the growth defect also in the *Δhbt2/Δhbt2* mutant suggesting that Hbt1 transports both 3-hydroxybenzoate and protocatechuate, although the native *HBT1* gene is differentially regulated in cells assimilating these substrates (Fig. 1). However, pPK5-*HBT2* does not functionally complement the *Δhbt1* mutation. We also found that neither *Δhbt1*/*Δhbt1* nor *Δhbt2*/*Δhbt2* cells transformed with the plasmid pPK5-*HBT3* can grow in media containing 3-hydroxybenzoate (*Δhbt1*/*Δhbt1*) or protocatechuate (*Δhbt2*/*Δhbt2*) suggesting that Hbt3 does not participate in the uptake of these substrates (Fig. 3).

**Figure 4:**
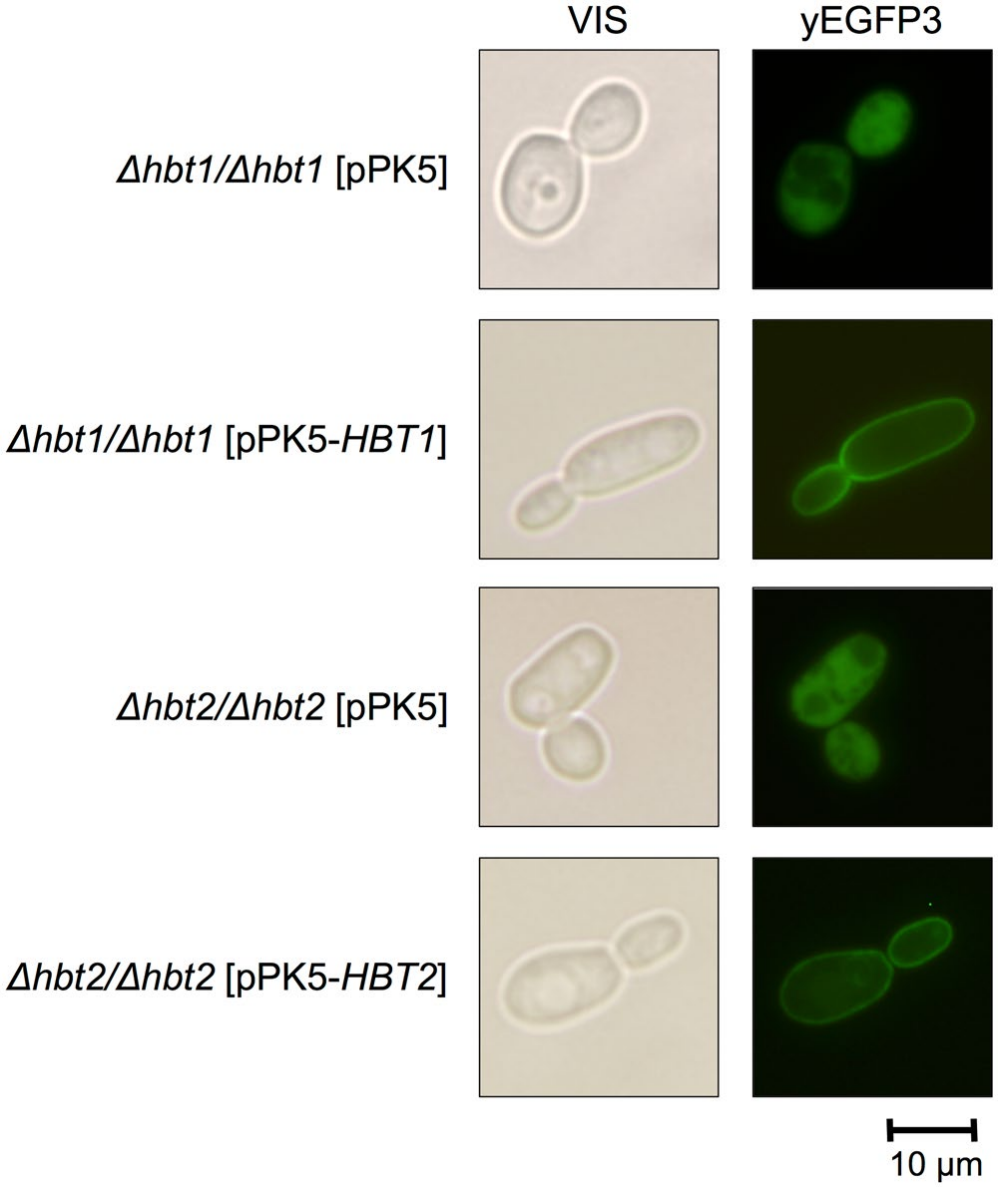
Intracellular localization of Hbt1 and Hbt2 proteins. The cells transformed with pPK5, pPK5-*HBT1* and pPK5-*HBT2* plasmids were grown overnight in SD + MPA medium at 28 °C, washed with water and the expression of yEGFP3-tagged proteins was induced for 2–4.5 hours by cultivation in SGal + MPA medium. The cells were examined by fluorescence microscopy (Olympus BX50).

The examination of the transformants by fluorescence microscopy showed that the fusion proteins Hbt1-yEGFP3 and Hbt2-yEGFP3 are localized on the cell surface, presumably in the plasma membrane (Fig. 4).

### Uptake of [^14^C]-labeled hydroxybenzoates

**Figure 5:**
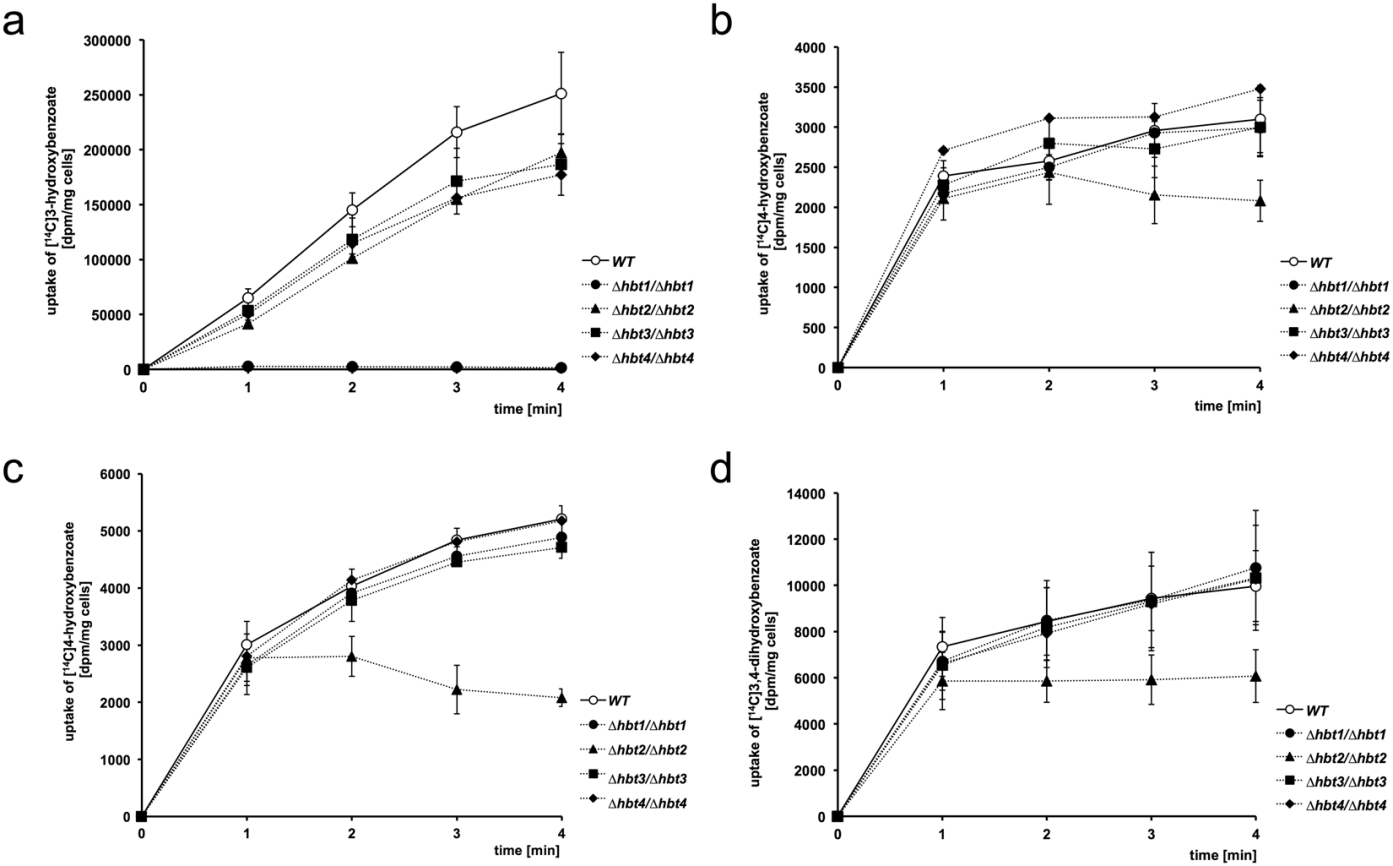
Uptake of [^14^C]-labeled hydroxybenzoates. *C. parapsilosis* cells CLIB214 (wild type) and mutants lacking individual Hbt carriers were grown in unbuffered synthetic media containing a hydroxyaromatic compound as a sole carbon source. The uptake assays were then performed as described in the Methods section. *C. parapsilosis* cells grown in S3OH (**a**) S4OH (**b**) and SHyd (**c**,**d**) medium were used in the uptake assays of [^14^C]3-hydroxybenzoate (**a**) [^14^C]4-hydroxybenzoate (**b**,**c**) and [^14^C]3,4-dihydroxybenzoate (**d**), respectively. The assays were performed in three independent experiments with two parallel measurements in each case (mean ± SEM).

To investigate the transport of hydroxybenzoates into *C. parapsilosis* cells, we analyzed the uptake of [^14^C]-labeled substrates (*i.e*. 3-hydroxybenzoate, 4-hydroxybenzoate and protocatechuate; Fig. 5). Our results showed that the wild type cells transport all three substrates, although the overall accumulated radioactivity in cells was substantially higher with [^14^C]3-hydroxybenzoate than [^14^C]4-hydroxybenzoate or [^14^C]3,4-dihydroxybenzoate. These differences may at least partially result from different levels of *HBT1-4* transcripts in the cells grown in S3OH, SHyd and S3,4diOH media (Fig. 1, Supplementary Table S2). Alternatively, corresponding transporters may exhibit different affinities to these substrates. The hydroxybenzoate uptake cannot be attributed to a simple diffusion as the assays were performed at pH 7.5, where about 99% of the substrate is present as a hydroxybenzoate anion that does not pass through the plasma membrane.

To examine if the transport of hydroxybenzoates is dependent on proton gradient, we treated the wild type cells with a protonophore (100 µM CCCP) prior addition of [^14^C]3-hydroxybenzoate to the uptake assay. We found that CCCP almost completely inhibits the substrate uptake (Table 1) thus confirming that the transport is driven by proton gradient across the plasma membrane.

**Table 1:** The uptake of [^14^C]3-hydroxybenzoate is blocked by protonophore and in the absence of Hbt1p. Results of the assay are expressed as the percentage (±SEM) of the wild type cells untreated with CCCP.

Strain	1 min	2 min	3 min	4 min
wild type	100	100	100	100
wild type + 100 µM CCCP	3.85 ± 0.38	4.83 ± 1.54	4.09 ± 0.74	3.68 ± 0.50
*∆hbt1/∆hbt1*	4.12 ± 1.66	1.68 ± 0.52	0.95 ± 0.08	0.56 ± 0.10

In contrast to the wild type cells, the uptake of [^14^C]3-hydroxybenzoate is almost completely abolished in the *Δhbt1/Δhbt1* mutant (Fig. 5a). This indicates that Hbt1p is the major carrier for this substrate. The impaired growth of this mutant in S3OH and S2,5diOH (Fig. 2) further suggests that Hbt1p can transport both 3-hydroxybenzoate as well as gentisate. The uptake of [^14^C]3-hydroxybenzoate is decreased by 21, 26 and 29% also in *Δhbt2/Δhbt2*, *Δhbt3/Δhbt3* and *Δhbt4/Δhbt4* cells, respectively. The reason for reduced transport capacity of these mutants remains unclear. However, it seems unlikely that Hbt2, Hbt3 and Hbt4 contribute to the uptake of 3-hydroxybenzoate as the cells lacking Hbt1 apparently do not transport this substrate. To analyze the kinetics of 3-hydroxybenzoate transport, we examined the uptake of this substrate in eight concentrations ranging from 0.5 to 150 µM. Our results showed that 3-hydroxybenzoate is transported into the wild type cells with *V*_*max*_ 0.63 ± 0.05 nmol/min/mg of dry weight and *K*_*m*_ 21.58 ± 6.58 µM (Supplementary Fig. S6) indicating that, similarly to bacterial AAHS permeases (*e.g*. GenK, MhbT and PcaK)^12–14^, Hbt1 is a high-affinity transporter.

The experiments with [^14^C]4-hydroxybenzoate and [^14^C]3,4-dihydroxybenzoate revealed that the uptake of both substrates is impaired in the *∆hbt2/∆hbt2* mutant but not in other strains (Fig. 5b–d). The growth defect of this strain in S4OH, S2,4diOH and S3,4diOH indicates that Hbt2p can transport all three hydroxybenzoates catabolized via the 3-oxoadipate pathway (Fig. 2).

In most cases, the MFS permeases transport their substrates into cells along with H^+^ ions^1, 44^. The hydroxyaromatic anion: H^+^ symport systems were demonstrated in bacteria^12, 45^ as well as in basidiomycetes *T. cutaneum* (phenolate)^20^ and *F. palustris* (vanillate)^22^. The amino acid sequence similarity of the Hbt proteins and bacterial AAHS transporters as well as the dependence of hydroxybenzoate uptake on the proton gradient led us to conclusion that *C. parapsilosis* Hbt proteins also function as proton symporters. We posit that Hbt1p and Hbt2p are involved in the uptake of hydroxybenzoates degraded via the gentisate and 3-oxoadipate pathway, respectively. The role of Hbt3p and Hbt4p remains elusive and will require further investigation.

### Phylogeny of *HBT* genes and the evolution of hydroxybenzoate catabolism

**Figure 6:**
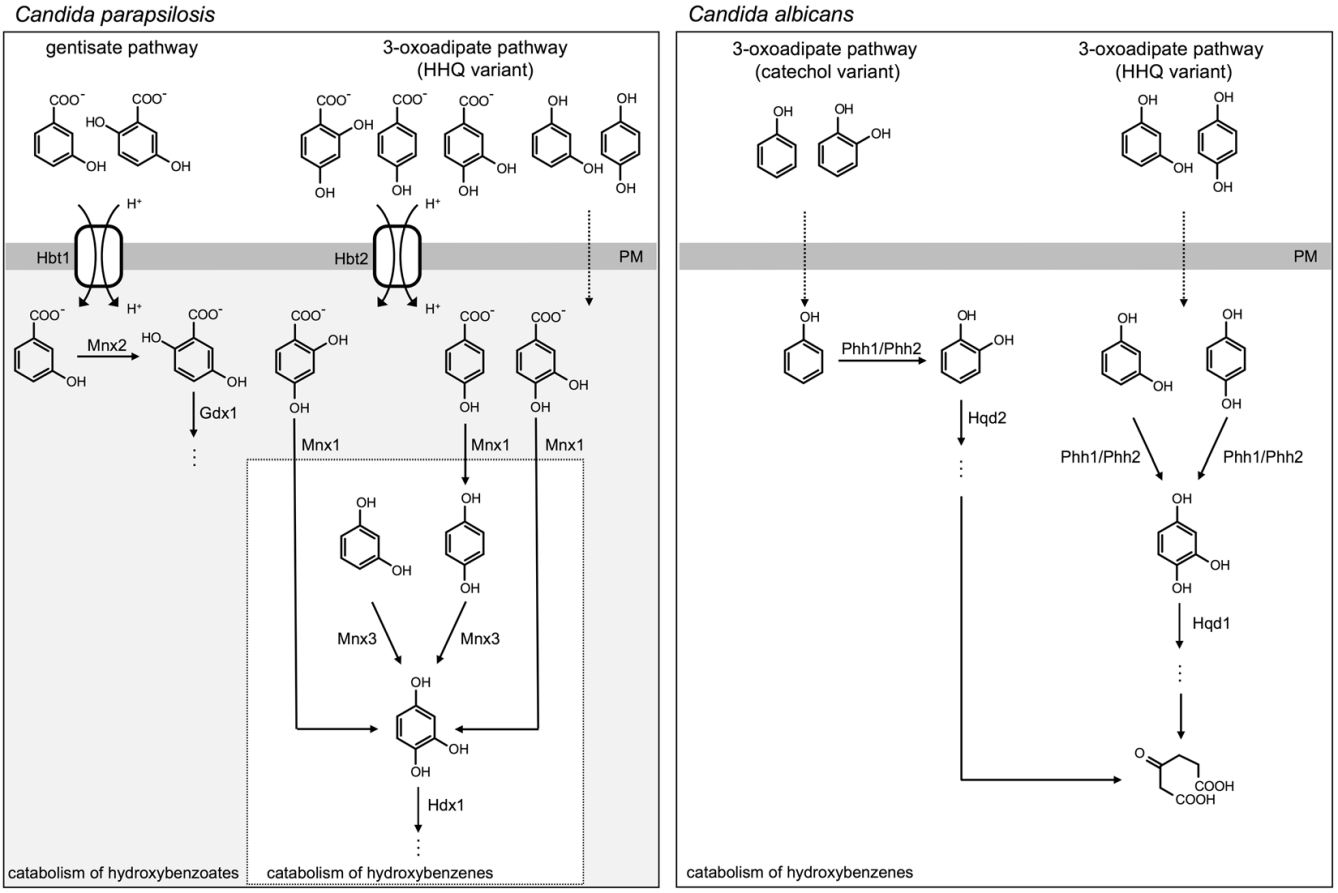
Degradation of hydroxybenzenes and hydroxybenzoates in the yeasts *C. parapsilosis* and *C. albicans*. The two *Candida* species differ in biochemical pathways involved in degradation of hydroxyaromatic substrates as well as in the transport systems for these compounds. While *C. parapsilosis* assimilates both hydroxybenzenes and hydroxybenzoates, *C. albicans* utilizes a wider range of hydroxybenzenes, but not hydroxybenzoates. Although the HHQ variant of the 3-oxoadipate pathway operates in both species, *C. albicans* lacks decarboxylating monooxygenase Mnx1 and hydroxybenzoate transporter Hbt2, which are present in *C. parapsilosis*. Mnx1 and Hbt2 are paralogous to Mnx2 and Hbt1, respectively, that are involved in the gentisate pathway. Note that *C. parapsilosis* proteins Mnx3 and Hdx1 are orthologous to *C. albicans* proteins Phh1/Phh2 and Hqd1, respectively.

Aromatic compounds derived from lignin in decaying plant tissues are catabolized via biochemical pathways operating in species from all three kingdoms of life (reviewed in ref. 46). In *C. parapsilosis*, the catabolism of hydroxybenzoates proceeds via the gentisate pathway and the hydroxyhydroquinone (HHQ) variant of the 3-oxoadipate pathway (Fig. 6). These pathways include activities of monooxygenases with broader substrate specificity (Mnx1, Mnx2, Mnx3) and dioxygenases (Hdx1, Gdx1) that open the aromatic ring of the catabolized compounds. Resulting products are then converted via several reactions to intermediates entering the central metabolism (*e.g*. tricarboxylic cycle). In contrast to *C. parapsilosis*, *C. albicans* lacks the gentisate pathway and possesses the catechol and HHQ variants of the 3-oxoadipate pathway^24–26, 38^. As *C. albicans* does not have a homolog of decarboxylating monooxygenase catalyzing the first step of the 3-oxoadipate pathway (*i.e*. Mnx1 in *C. parapsilosis*) and lacks homologs of the Hbt transporters, it can utilize hydroxybenzenes, but not hydroxybenzoates (Fig. 6).

The difference between the two *Candida* species belonging to the CTG clade of Saccharomycotina could result from either a downgrade or an upgrade of corresponding metabolic pathways. In the first scenario, a common ancestor of the CTG clade species possessed the two branches of the 3-oxoadipate pathway as well as the gentisate pathway and it could catabolize both hydroxybenzoates and hydroxybenzenes. The gentisate pathway gene cluster and the genes coding for decarboxylating monooxygenase (Mnx1) and hydroxybenzoate transporters have been lost in the *C. albicans* lineage causing its inability to assimilate hydroxybenzoates. On the other hand, the *C. parapsilosis* lineage lost the genes for the catechol branch of the 3-oxoadipate pathway. Alternatively, an ancestor of both species possessed the catechol branch and a shorter version of the HHQ variant of the 3-oxoadipate pathway allowing the degradation of hydroxybenzenes (*i.e*. as it occurs in *C. albicans*) and the gentisate pathway. The genes encoding the enzymes of the latter pathway and the catechol variant of the 3-oxoadipate pathway have been lost in the lineages leading to *C. albicans* and *C. parapsilosis*, respectively. In the *C. parapsilosis* lineage, the duplications of *HBT1* and *MNX2* would generate *HBT2* and *MNX1*, respectively, and led to an upgrade of the HHQ variant of the 3-oxoadipate pathway to allow the uptake and decarboxylation of hydroxybenzoates.

**Figure 7:**
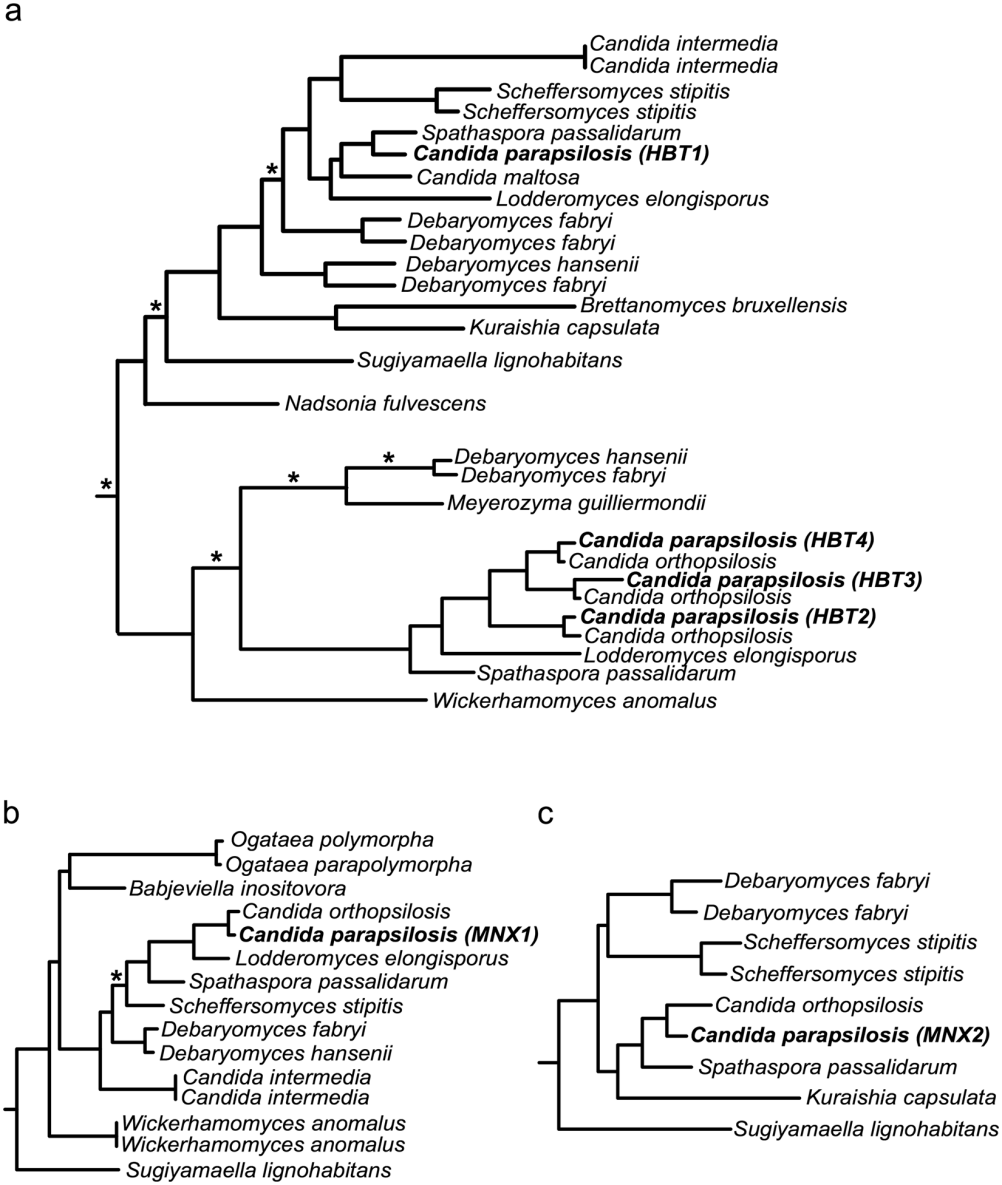
Relevant partitions of the reconstructed phylogenies of the genes *HBT1* and *HBT2* (**a**) *MNX1* (**b**) and *MNX2* (**c**). Every tree shows the largest monophyletic clade containing the seed sequence (*HBT1*, *MNX1* and *MNX2*, respectively) and their Saccharomycotina homologs. The subtrees have been rooted at the closest non-Saccharomycotina homologs. The branches with low support (aLRT < 0.5) are shown with an asterisk.

To examine these possibilities, we performed phylogenetic analysis of hydroxybenzoate transporters and their homologs (Fig. 7, see Methods). We found that the *HBT1* orthologs are widespread in Pezizomycotina (*e.g. Aspergillus* and *Penicillium* species), but that their distribution among Saccharomycotina is very sparse. In the latter subphylum, the orthologs were identified in several species of the CTG clade (*i.e. Candida maltosa, Debaryomyces hansenii*, *Debaryomyces fabryi*, *Lodderomyces elongisporus*, *Scheffersomyces stipitis*, *Spathaspora passalidarum*) as well as in species classified outside of this phylogenetic branch (*i.e. Brettanomyces bruxellensis, Kuraishia capsulata, Nadsonia fulvescens*, *Sugiyamaella lignohabitans*). *HBT2* orthologs show a more sparse distribution which is almost restricted to the CTG clade, with the sole exception of *Wickerhamomyces anomalus*. The distribution within the CTG clade only partially overlaps with that of Hbt1, being present in several common species (*i.e. C. parapsilosis*, *D. hansenii, D. fabryi, L. elongisporus, S. passalidarum*) and some additional species (*C. orthopsilosis, M. guilliermondi*). Importantly, our comparative and phylogenetic analyses suggest that Hbt1 and Hbt2 transporters show a high level of sequence divergence, and with the associations of these two clades receiving only weak phylogenetic support. Considering our obtained topology (Fig. 7a), the most parsimonious phylogenetic scenario suggests that Hbt1 and Hbt2 diverged within the Saccharomycotina but much before the origin of the CTG clade. Differential losses from a common ancestor would have resulted in the current sparse distribution. Finally, our analysis identified that the closer paralogs to *HBT2* (*i.e. HBT3* and *HBT4*) emerged from a specific duplication in the *C. parapsilosis* complex species (*i.e. C. orthopsilosis* and *C. parapsilosis*).

Vertical descent from a common ancestor of Saccharomycotina was proposed for evolution of the metabolic gene cluster encoding the gentisate and 3-oxoadipate pathway enzymes^38, 39^. Phylogenetic distribution of the *HBT* paralogs correlates with the ability to grow on hydroxybenzoates. Hence, the duplication of *HBT1* followed by functional specialization of *HBT2* could extend the range of hydroxybenzoate substrates metabolized via the 3-oxoadipate pathway, supporting the upgrading scenario. However, our analysis indicates that this occurred a long time ago, before the divergence of the CTG clade from other Saccharomycotina, and that subsequent loss in some lineages resulted in the secondary loss of this ability.

As mentioned above, the upgrade of the 3-oxoadipate pathway requires a decarboxylation of a hydroxybenzoate substrate. In *C. parapsilosis*, this reaction is catalyzed by 4-hydroxybenzoate 1-hydroxylase (Mnx1), which has broad substrate specificity and utilizes 4-hydroxybenzoate, 2,4-dihydroxybenzoate and protocatechuate^26, 47^. This enzyme has a distant paralog Mnx2 (3-hydroxybenzoate 6-hydroxylase) catalyzing the first reaction of the gentisate pathway. The phylogenetic analysis of Mnx1 and Mnx2 indicates that their emergence from a common ancestor is also evolutionarily ancient and occurred before the divergence of the CTG clade from a Saccharomycotina ancestor. Based on the phylogenies of Hbt1-4 and Mnx1-2 proteins (Fig. 7) we assume that the ancestor possessed the gentisate pathway as well as the longer version of the 3-oxoadipate pathway, but had limited capacity for the hydroxybenzoate uptake as it possessed only the Hbt1 transporter. This limitation was overcome by the *HBT1* gene duplication and subsequent specialization of the Hbt2 transporter. Many subsequent lineages have lost either of the pathways, given rise to their sparse distributions with little overlap that we see today. Thus, we propose a combined model, implying one ancient upgrade followed by multiple independent downgrades. Of note, *HBT1* duplications seem to be common. However, the phylogenetic analysis indicates that these paralogs emerged independently of *HBT2*.

## Conclusions

The yeast *C. parapsilosis* possesses MFS carriers Hbt1 and Hbt2 mediating the inducible proton driven transport system for hydroxybenzoates catabolized via the gentisate and 3-oxoadipate pathway, respectively. These transporters are functionally related to bacterial AAHS permeases and represent the first identified eukaryotic members of this family. Functional characterization of these carriers may contribute to exploration of yeast species in bioremediation of environments contaminated with toxic aromatic pollutants and utilization of compounds derived from lignin and decaying plant tissues.

## Methods

### Yeast cultivations

*C. parapsilosis* CLIB214 (identical to CBS604, the wild type strain) and its mutant derivatives were cultivated in synthetic and complex media listed in Table 2. The sensitivity to acidic and alkaline conditions was tested in YPD media with pH ranging from 4 to 8. pH of the respective media was adjusted using McIlvaine (citrate-phosphate) buffer. The temperature sensitivity was examined in YPD medium at 20, 30 and 37 °C for 48 hours. The growth kinetics was assessed in liquid YPD medium for 24 hours at 30 °C with hourly measurements at OD_600_. The viability was also monitored in the presence of various stressors applied in serial twofold dilutions in six steps. The following stressors were used: calcofluor white (0.1 mg/ml), congo red (0.1 mg/ml), caffeine (0–50 mM), H_2_O_2_ (7.5 mM), sodium dodecyl sulfate (SDS, 0.1% (w/v)) and hygromycin B (0.0156 mg/ml). The survival relative to the stressor-free control was monitored by measurements at OD_600_. The formation of pseudohyphae was analyzed in DMEM + FSB medium after 24 and 48 hours at 37 °C. In experiments aimed at the functional analysis of *HBT* genes, the cells were grown at 28 °C in synthetic media containing a hydroxyaromatic compound, glucose, galactose, glycerol or ethanol as a sole carbon source (Table 2). Hydroxyaromatic compounds were dissolved in dimethyl sulfoxide (DMSO) as 0.5 M stocks. Where indicated, pH of the medium was adjusted to 7.5 using 100 mM Tris-HCl prior addition of a carbon source. For solid media, agar was added to 2% (w/v).

**Table 2:** Cultivation media.

Medium	Composition
DMEM + FSB	Dulbecco’s Modified Eagle’s Medium (Lonza), 10% (w/v) heat-inactivated fetal bovine serum (EuroClone)
SD	0.67% (w/v) yeast nitrogen base w/o amino acids (Difco), 2% (w/v) glucose
SD + MPA	0.67% (w/v) yeast nitrogen base w/o amino acids (Difco), 2% (w/v) glucose, 200 μg/ml mycophenolic acid (Sigma-Aldrich)
SD_1%_	0.67% (w/v) yeast nitrogen base w/o amino acids (Difco), 1% (w/v) glucose
SD_1%_ + FBS	0.67% (w/v) yeast nitrogen base w/o amino acids (Difco), 1% (w/v) glucose, 10% (v/v) heat-inactivated fetal bovine serum (EuroClone)
S3OH	0.67% (w/v) yeast nitrogen base w/o amino acids (Difco), 10 mM 3-hydroxybenzoate
S4OH	0.67% (w/v) yeast nitrogen base w/o amino acids (Difco), 10 mM 4-hydroxybenzoate
S2,4diOH	0.67% (w/v) yeast nitrogen base w/o amino acids (Difco), 10 mM 2,4-dihydroxybenzoate (β-resorcylate)
S2,5diOH	0.67% (w/v) yeast nitrogen base w/o amino acids (Difco), 10 mM 2,5-dihydroxybenzoate (gentisate)
S3,4diOH	0.67% (w/v) yeast nitrogen base w/o amino acids (Difco), 10 mM 3,4-dihydroxybenzoate (protocatechuate)
SHyd	0.67% (w/v) yeast nitrogen base w/o amino acids (Difco), 10 mM hydroquinone
SRes	0.67% (w/v) yeast nitrogen base w/o amino acids (Difco), 10 mM resorcinol
SEtOH	0.67% (w/v) yeast nitrogen base w/o amino acids (Difco), 2% (v/v) ethanol
SGal + MPA	0.67% (w/v) yeast nitrogen base w/o amino acids (Difco), 2% (w/v) galactose, 200 μg/ml mycophenolic acid
SGly	0.67% (w/v) yeast nitrogen base w/o amino acids (Difco), 3% (w/v) glycerol
YCB + BSA	1.17% (w/v) yeast carbon base (Difco), 2% (w/v) bovine serum albumin
YPD	1% (w/v) yeast extract, 1% (w/v) peptone, 0.5% (w/v) glucose

### Preparation of knockout strains

The mutants lacking an individual *HBT* gene were generated using a previously established method, adapted for *C. parapsilosis* ^48, 49^. Briefly, the disruption of a gene was achieved via auxotrophy complementation of the double auxotrophic strain named CPL2H1 (*C. parapsilosis his* ^−^/*leu* ^−^ derived from CLIB214). Deletion constructions contained the upstream (UpFw primer 1 and UpRev primer 3; Supplementary Table S6) and downstream (DownFw primer 4 and DownRev primer 6) homologous regions of the target open reading frame (ORF) and either *Candida dubliniensis HIS1* or *Candida maltosa LEU2* sequences as selection markers. Coding sequences of *HIS1* and *LEU2* were amplified from the plasmids pSN52 and pSN40, respectively^48, 49^, using the primer 2 and 5. Joining of the amplified products was achieved using polymerase chain reaction (PCR). Deletion cassettes were transformed into CPL2H1 strain and the transformants were plated onto selective media. Obtained heterozygous and homozygous mutant strains were verified by colony PCR using the primers specific for both the marker sequences and the outside of the integration sites at both the upstream and downstream homologous regions. Mutant strains were further tested for the gene expression using quantitative PCR (qPCR).

### Gene expression analysis

Total cellular RNA was isolated from the culture of the wild type strain grown in synthetic media with appropriate carbon source by the phenol-chloroform extraction protocol^50^ and the RNA preparations were treated with RNase-free DNase I (New England Biolabs) according to the manufacturer’s instructions. The RNA-seq and real-time qPCR analyses were performed as described previously^39^. The sequencing reads have been deposited to Short Read Archive (PRJEB1707). The gene-specific primers used for qPCR assays are shown in Supplementary Table S6.

### Plasmid constructs

The *C. parapsilosis* sequences coding for Hbt1, Hbt2 and Hbt3 proteins were amplified by PCR using the gene specific primers (Supplementary Table S6) and the template from the genomic DNA of the wild type strain. The PCR products containing the ORFs without the termination codon, plus 700 nucleotides upstream of the initiation codon, were inserted into the *Sal*I site of the pPK5 vector^51^ using the Gibson assembly cloning kit (New England Biolabs). The cloned genes containing native promoters are placed downstream of the *GAL1* promoter in the resulting plasmid constructs.

### Functional complementation of Δ*hbt* mutants and fluorescence microscopy

Plasmid DNAs were introduced into *Δhbt* cells by electroporation^52^ and transformants were selected on media containing mycophenolic acid (SD + MPA). The utilization of hydroxybenzoates was tested in synthetic media containing glucose or a hydroxybenzoate as a sole carbon source. The cells transformed with the vector pPK5 were used as a negative control. For intracellular localization of Hbt proteins C-terminally tagged with yeast-enhanced green fluorescent protein 3 (yEGFP3), the transformants were cultivated overnight in SD + MPA medium at 28 °C, washed with water and the expression of fusion proteins was induced for 2–4.5 hours in SGal + MPA medium. Intracellular localization of Hbt1-yEGFP3 and Hbt2-yEGFP3 was investigated by fluorescence microscopy using BX50 microscope equipped with the appropriate filter set and digital camera DP70 (Olympus Optical).

### Hydroxybenzoate uptake assays

*C. parapsilosis* cells were grown in synthetic media supplemented with hydroxyaromatic substrate (10 mM) as a sole carbon source (*i.e*. S3OH, S4OH and SHyd for the uptake of [^14^C]3-hydroxybenzoate, [^14^C]4-hydroxybenzoate and [^14^C]3,4-dihydroxybenzoate, respectively) till late exponential phase and harvested by centrifugation (10 min, 3000 *g* at 4 °C). The pellet was washed once with ice-cold water and once with the assay buffer (50 mM Tris-HCl pH 7.5) and then resuspended in the same buffer at an OD_600_ = 90. For each measurement, a 60 μl aliquot of cell suspension was used. The uptake assays were performed in the assay buffer at 28 °C. Labeled substrates (*i.e*. [carboxyl-^14^C]3-hydroxybenzoate (55 mCi/mmol), [carboxyl-^14^C]4-hydroxybenzoate (55 mCi/mmol) and [carboxyl-^14^C]3,4-dihydroxybenzoate (55 mCi/mmol)) were purchased from American Radiolabeled Chemicals, Inc. (St. Louis, MO). Uptake was initiated by addition of a [^14^C]-labeled compound to a final concentration 50 μM. Aliquots (75 μl) were taken from the incubation mixture (364 μl) at timed intervals, immediately filtered through cellulose membranes (0.45-μm pore size; MF-Millipore) and washed twice with 4 ml ice-cold assay buffer. The amount of radioactivity accumulated in the cells was determined with a scintillation counter Tri-Carb 2900 TR (Perkin Elmer). The uptake activity was expressed as disintegrations per minute (dpm) per mg of cells (dry weight). The effect of a protonophore on the uptake of [^14^C]3-hydroxybenzoate was tested in cells treated for two minutes with 100 µM carbonyl cyanide *m*-chlorophenyl hydrazone (CCCP) prior addition of [^14^C]-labeled substrate to the uptake assay. Apparent *K*_*m*_ and *V*_max_ values for the 3-hydroxybenzoate uptake were obtained essentially as described for bacterial GenK transporter^14^.

### Bioinformatic analyses

The following databases were used to identify and analyze the gene and protein sequences: *Candida* Genome Database (http://www.candidagenome.org/)^53^, *Candida* Gene Order Browser (http://cgob3.ucd.ie/)^54, 55^; Pfam database (http://pfam.xfam.org/)^56^, SMART (http://smart.embl-heidelberg.de/)^57^, UniProt (http://www.uniprot.org/)^58^ and Transporter Classification Database (http://www.tcdb.org/)^7^. Transmembrane helices were predicted by the TMHMM server v 2.0 (http://www.cbs.dtu.dk/services/TMHMM/)^59^ and the secondary structures of transporter proteins were visualized using Protter (http://wlab.ethz.ch/protter/)^60^. Amino acid sequences were aligned using the MAFFT algorithm implemented in the Geneious package v. 5.6.6 (Biomatters) and the alignments were further processed in the Jalview workbench^61^.

### Phylogenetic analysis

Evolutionary histories of the genes considered were first visually inspected using the Maximum Likelihood phylogenies available in PhylomeDB^62^. Then individual phylogenies were reconstructed using these proteins and their closest blast hits in NCBI non-redundant database searched as of January 2017. The first 100 blast hits were used for *MNX1* and *MNX2* genes. For *HBT1* and *HBT2* this procedure did not render both genes within the first 100 hits of each other, but both lists contained common proteins. We thus performed the analysis of the combination of both lists. In brief, phylogenies were reconstructed as follows: protein sequences of the hits that passed a threshold of similarity (e-value < 10^−5^) and coverage (>33% aligned over the query sequence), were aligned with MUSCLE^63^ with default parameters, trimmed with trimAl v1.4^64^ to eliminate alignment columns with more than 50% gaps. A Maximum Likelihood phylogenetic reconstruction was performed with PhyML v3^65^, using the LG model and approximating four rate categories and the fraction of invariable sites from the data. Then monophyletic clades containing the seed proteins and other Saccharomycotina species were selected. Duplicated sequences and pseudogenes were removed. Subtrees are shown in Fig. 7. Whole trees (in Newick format) are provided in Supplementary Files S1–S3.

## Electronic supplementary material

Supplementary Information

## References

[1] Pao, S. S., Paulsen, I. T. & Saier, M. H. Jr. Major facilitator superfamily. *Microbiol. Mol. Biol. Rev*. **62**, 1–34 (1998). 10.1128/mmbr.62.1.1-34.1998 PMC98904 9529885

[2] Saier MH (1999). The major facilitator superfamily. J. Mol. Microbiol. Biotechnol..

[3] Chang AB, Lin R, Keith Studley W, Tran CV, Saier MH (2004). Phylogeny as a guide to structure and function of membrane transport proteins. Mol. Membr. Biol..

[4] Law CJ, Maloney PC, Wang DN (2008). Ins and outs of major facilitator superfamily antiporters. Annu. Rev. Microbiol..

[5] Reddy VS, Shlykov MA, Castillo R, Sun EI, Saier MH (2012). The major facilitator superfamily (MFS) revisited. FEBS J..

[6] Yan N (2013). Structural advances for the major facilitator superfamily (MFS) transporters. Trends Biochem. Sci..

[7] Saier MH (2016). The Transporter Classification Database (TCDB): recent advances. Nucleic Acids Res..

[8] Nomura Y, Nakagawa M, Ogawa N, Harashima S, Oshima Y (1992). Genes in PHT plasmid encoding the initial degradation pathway of phthalate in *Pseudomonas putida*. J. Ferment. Bioeng..

[9] Chang HK, Zylstra GJ (1999). Characterization of the phthalate permease OphD from *Burkholderia cepacia* ATCC 17616. J. Bacteriol..

[10] Chang HK, Dennis JJ, Zylstra GJ (2009). Involvement of two transport systems and a specific porin in the uptake of phthalate by *Burkholderia* spp. J. Bacteriol..

[11] Collier LS, Nichols NN, Neidle EL (1997). *benK* encodes a hydrophobic permease-like protein involved in benzoate degradation by *Acinetobacter* sp. strain ADP1. J. Bacteriol..

[12] Nichols NN, Harwood CS (1997). PcaK, a high-affinity permease for the aromatic compounds 4-hydroxybenzoate and protocatechuate from *Pseudomonas putida*. J. Bacteriol..

[13] Xu Y (2012). MhbT is a specific transporter for 3-hydroxybenzoate uptake by Gram-negative bacteria. Appl. Environ. Microbiol..

[14] Xu Y, Wang SH, Chao HJ, Liu SJ, Zhou NY (2012). Biochemical and molecular characterization of the gentisate transporter GenK in *Corynebacterium glutamicum*. PLoS One.

[15] Karimian M, Ornston LN (1981). Participation of the beta-ketoadipate transport system in chemotaxis. J. Gen. Microbiol..

[16] Saint CP, Romas P (1996). 4-Methylphthalate catabolism in *Burkholderia (Pseudomonas) cepacia* Pc701: a gene encoding a phthalate-specific permease forms part of a novel gene cluster. Microbiology.

[17] Whipp MJ, Camakaris H, Pittard AJ (1998). Cloning and analysis of the *shiA* gene, which encodes the shikimate transport system of *Escherichia coli* K-12. Gene.

[18] Neidle EL (1991). Nucleotide sequences of the *Acinetobacter calcoaceticus benABC* genes for benzoate 1,2-dioxygenase reveal evolutionary relationships among multicomponent oxygenases. J. Bacteriol..

[19] Clark TJ, Momany C, Neidle EL (2002). The *benPK* operon, proposed to play a role in transport, is part of a regulon for benzoate catabolism in *Acinetobacter* sp. strain ADP1. Microbiology.

[20] Mortberg M, Neujahr HY (1985). Uptake of phenol by *Trichosporon cutaneum*. J. Bacteriol..

[21] Mortberg M, Spanning A, Neujahr HY (1988). Induction of high-affinity phenol uptake in glycerol-grown *Trichosporon cutaneum*. J. Bacteriol..

[22] Shimizu M, Kobayashi Y, Tanaka H, Wariishi H (2005). Transportation mechanism for vanillin uptake through fungal plasma membrane. Appl. Microbiol. Biotechnol..

[23] Gopal E (2007). Transport of nicotinate and structurally related compounds by human SMCT1 (SLC5A8) and its relevance to drug transport in the mammalian intestinal tract. Pharm. Res..

[24] Middelhoven WJ (1993). Catabolism of benzene compounds by ascomycetous and basidiomycetous yeasts and yeastlike fungi. A literature review and an experimental approach. Antonie van Leeuwenhoek.

[25] Middelhoven WJ, Coenen A, Kraakman B, Sollewijn Gelpke MD (1992). Degradation of some phenols and hydroxybenzoates by the imperfect ascomycetous yeasts *Candida parapsilosis* and *Arxula adeninivorans*: evidence for an operative gentisate pathway. Antonie van Leeuwenhoek.

[26] Holesova Z (2011). Gentisate and 3-oxoadipate pathways in the yeast *Candida parapsilosis*: identification and functional analysis of the genes coding for 3-hydroxybenzoate 6-hydroxylase and 4-hydroxybenzoate 1-hydroxylase. Microbiology.

[27] Dujon B (2004). Genome evolution in yeasts. Nature.

[28] Jones T (2004). The diploid genome sequence of *Candida albicans*. Proc. Natl. Acad. Sci. USA.

[29] Jeffries TW (2007). Genome sequence of the lignocellulose-bioconverting and xylose-fermenting yeast *Pichia stipitis*. Nat. Biotechnol..

[30] Butler G (2009). Evolution of pathogenicity and sexual reproduction in eight *Candida* genomes. Nature.

[31] Jackson AP (2009). Comparative genomics of the fungal pathogens *Candida dubliniensis* and *Candida albicans*. Genome Res..

[32] Riccombeni A, Vidanes G, Proux-Wéra E, Wolfe KH, Butler G (2012). Sequence and analysis of the genome of the pathogenic yeast *Candida orthopsilosis*. PLoS One.

[33] Pryszcz LP, Nemeth T, Gacser A, Gabaldon T (2014). Genome comparison of *Candida orthopsilosis* clinical strains reveals the existence of hybrids between two distinct subspecies. Genome Biol. Evol..

[34] Pryszcz LP (2015). The genomic aftermath of hybridization in the opportunistic pathogen *Candida metapsilosis*. PLoS Genet..

[35] Gaur M (2008). MFS transportome of the human pathogenic yeast *Candida albicans*. BMC Genomics.

[36] Costa C, Dias PJ, Sá-Correia I, Teixeira MC (2014). MFS multidrug transporters in pathogenic fungi: do they have real clinical impact?. Front. Physiol..

[37] Dias PJ, Sa-Correia I (2014). Phylogenetic and syntenic analyses of the 12-spanner drug:H(+) antiporter family 1 (DHA1) in pathogenic *Candida* species: evolution of *MDR1* and *FLU1* genes. Genomics.

[38] Gerecova G (2015). Metabolic gene clusters encoding the enzymes of two branches of the 3-oxoadipate pathway in the pathogenic yeast *Candida albicans*. FEMS Yeast Res..

[39] Zeman I (2016). Mitochondrial carriers link the catabolism of hydroxyaromatic compounds to the central metabolism in *Candida parapsilosis*. G3 (Bethesda).

[40] Fan J, Chaturvedi V, Shen SH (2002). Identification and phylogenetic analysis of a glucose transporter gene family from the human pathogenic yeast *Candida albicans*. J. Mol. Evol..

[41] Brown V, Sexton JA, Johnston M (2006). A glucose sensor in *Candida albicans*. Eukaryot. Cell.

[42] Ditty JL, Harwood CS (1999). Conserved cytoplasmic loops are important for both the transport and chemotaxis functions of PcaK, a protein from *Pseudomonas putida* with 12 membrane-spanning regions. J. Bacteriol..

[43] Ditty JL, Harwood CS (2002). Charged amino acids conserved in the aromatic acid/H^+^ symporter family of permeases are required for 4-hydroxybenzoate transport by PcaK from *Pseudomonas putida*. J. Bacteriol..

[44] Yan N (2015). Structural biology of the major facilitator superfamily transporters. Annu. Rev. Biophys..

[45] Xu Y, Chen B, Chao H, Zhou NY (2013). mhpT encodes an active transporter involved in 3-(3-hydroxyphenyl)propionate catabolism by *Escherichia coli* K-12. Appl. Environ. Microbiol..

[46] Fuchs G, Boll M, Heider J (2011). Microbial degradation of aromatic compounds - from one strategy to four. Nat. Rev. Microbiol..

[47] Eppink MH, Boeren SA, Vervoort J, van Berkel WJ (1997). Purification and properties of 4-hydroxybenzoate 1-hydroxylase (decarboxylating), a novel flavin adenine dinucleotide-dependent monooxygenase from *Candida parapsilosis* CBS604. J Bacteriol.

[48] Noble SM, Johnson AD (2005). Strains and strategies for large-scale gene deletion studies of the diploid human fungal pathogen *Candida albicans*. Eukaryot. Cell.

[49] Holland LM (2014). Comparative phenotypic analysis of the major fungal pathogens *Candida parapsilosis* and *Candida albicans*. PLoS Pathog..

[50] Cross FR, Tinkelenberg AH (1991). A potential positive feedback loop controlling *CLN1* and *CLN2* gene expression at the start of the yeast cell cycle. Cell.

[51] Kosa P, Gavenciakova B, Nosek J (2007). Development of a set of plasmid vectors for genetic manipulations of the pathogenic yeast *Candida parapsilosis*. Gene.

[52] Zemanova J, Nosek J, Tomaska L (2004). High-efficiency transformation of the pathogenic yeast *Candida parapsilosis*. Curr. Genet..

[53] Inglis DO (2012). The *Candida* Genome Database incorporates multiple *Candida* species: multispecies search and analysis tools with curated gene and protein information for *Candida albicans* and *Candida glabrata*. Nucleic Acids Res..

[54] Fitzpatrick DA, O’Gaora P, Byrne KP, Butler G (2010). Analysis of gene evolution and metabolic pathways using the *Candida* Gene Order Browser. BMC Genomics.

[55] Maguire SL (2013). Comparative genome analysis and gene finding in *Candida* species using CGOB. Mol. Biol. Evol..

[56] Finn RD (2016). The Pfam protein families database: towards a more sustainable future. Nucleic Acids Res..

[57] Letunic I, Doerks T, Bork P (2015). SMART: recent updates, new developments and status in 2015. Nucleic Acids Res..

[58] The UniProt Consortium. UniProt: a hub for protein information. *Nucleic Acids Res*. **43**, D204–D212; doi:10.1093/nar/gku989 (2015). 10.1093/nar/gku989 PMC4384041 25348405

[59] Krogh A, Larsson B, von Heijne G, Sonnhammer ELL (2001). Predicting transmembrane protein topology with a hidden Markov model: Application to complete genomes. J. Mol. Biol..

[60] Omasits U, Ahrens CH, Müller S, Wollscheid B (2014). Protter: interactive protein feature visualization and integration with experimental proteomic data. Bioinformatics.

[61] Waterhouse AM, Procter JB, Martin DMA, Clamp M, Barton GJ (2009). Jalview Version 2-a multiple sequence alignment editor and analysis workbench. Bioinformatics.

[62] Huerta-Cepas J, Capella-Gutiérrez S, Pryszcz LP, Marcet-Houben M, Gabaldón T (2014). (2014) PhylomeDB v4: zooming into the plurality of evolutionary histories of a genome. Nucleic Acids Res..

[63] Edgar RC (2004). MUSCLE: a multiple sequence alignment method with reduced time and space complexity. BMC Bioinformatics.

[64] Capella-Gutiérrez S, Silla-Martínez JM, Gabaldón T (2009). trimAl: a tool for automated alignment trimming in large-scale phylogenetic analyses. Bioinformatics.

[65] Guindon S, Gascuel O (2003). A simple, fast, and accurate algorithm to estimate large phylogenies by maximum likelihood. Syst. Biol..

