# Supplementary material for: Eukaryotic transporters for hydroxyderivatives of benzoic acid
Source: Sci Rep. 2017 Aug 21;7:8998. doi: 10.1038/s41598-017-09408-6 (PMC5566891; doi:10.1038/s41598-017-09408-6)
Supplement: Supplementary file 1 — Supplementary Information [file 41598_2017_9408_MOESM1_ESM.pdf]

Eukaryotic transporters for hydroxyderivatives of benzoic acid

Andrea Cillingová[1,#], Igor Zeman[1,#], Renáta Tóth[2,#], Martina Neboháčová[1,#], Ivana Dunčková[1], Mária Hölcová[1], Michaela Jakúbková[1], Gabriela Gérecová[1,3], Leszek P. Pryszcz[4,5], Ľubomír Tomáška[1], Toni Gabaldón[4,6,7], Attila Gácser[2] and Jozef Nosek[1,*]

[1] Departments of Biochemistry and Genetics, Comenius University in Bratislava, Faculty of Natural Sciences, Ilkovičova 6, 842 15 Bratislava, Slovak Republic
[2] Department of Microbiology, University of Szeged, Szeged, Közép fasor 52, H-6726 Szeged, Hungary
[3] Present address: Department of Biochemistry and Cell Biology, Max F. Perutz Laboratories, University of Vienna, Dr. Bohr Gasse 9, 1030 Vienna, Austria
[4] Bioinformatics and Genomics Programme, Centre for Genomic Regulation, Doctor Aiguader 88, 08003 Barcelona, Spain
[5] Present address: International Institute of Molecular and Cell Biology in Warsaw, 4 Trojdena Street, 02-109 Warsaw, Poland
[6] Departament de Ciències Experimentals I de la Salut, Universitat Pompeu Fabra, 08003 Barcelona, Spain
[7] Institució Catalana de Recerca i Estudis Avançats, Pg. Lluís Companys 23, 08010 Barcelona, Spain

Supplementary Information

Supplementary Table S1.
Results of the BlastP searches for C. parapsilosis homologs of the aromatic compounds transporters.

**Query**<br>**Superfamily / Family / Protein**	**Uniprot acc.no.**	**Hits below < 1e-20** [Best hit]	**E-value**
**Superfamily: ABC**			
OphF	C0LZR7_BURCE	[CPAR2_210710]	9.5e-01
OphG	C0LZR8_BURCE	[CPAR2_109210]	3.6e-01
OphH	C0LZR9_BURCE	CPAR2_801330	6.0e-20
**Superfamily: MFS**			
**Family: ACS**			
OphD	Q9RPP3_BURCE	**CPAR2_802720**	**2.0e-35**
		**CPAR2_802710**	**3.0e-34**
		**CPAR2_802700**	**6.0e-34**
		**CPAR2_802690**	**2.0e-30**
		CPAR2_207250	3.0e-24
		CPAR2_209690	5.0e-24
		CPAR2_207260	2.0e-23
		CPAR2_207270	1.0e-22
		CPAR2_209700	2.0e-22
		CPAR2_502820	3.0e-22
		CPAR2_100300	3.0e-22
		CPAR2_209680	2.0e-21
		CPAR2_702960	5.0e-20
		CPAR2_702950	5.0e-20
OphP	C0LZS0_BURCE	[CPAR2_104420]	5.0e+00
Pth1	PHT1_PSEPU	**CPAR2_802710**	**2.0e-22**
		**CPAR2_802700**	**4.0e-22**
		**CPAR2_802720**	**2.0e-21**
**Family: AAHS**			
BenK	E0X724_PSEPU	[CPAR2_109920]	7.0e-11
		[CPAR2_402050]	7.0e-11
GenK	GENK_CORGL	[CPAR2_407640]	1.0e-10
		[CPAR2_100490]	1.0e-10
MhbT	MHBT_KLEOX	[CPAR2_802600]	2.0e-09
PcaK	PCAK_PSEPU	[CPAR2_802600]	3.0e-10
**Family: MHS**			
MopB	Q45082_BURCE	[CPAR2_407640]	3.0e-11
PcaT	G8PXG0_PSEFL	[CPAR2_100490]	8.0e-08
ShiA	SHIA_ECOLI	[CPAR2_400100]	8.0e-09
**Superfamily: APC**			
BenE	BENE_ACIAD	[CPAR2_802240]	1.7e+00
**Superfamily: OMPP (porin)**			
BenP	Q9XC30_ACIAD	[CPAR2_211110]	9.1e-01

Supplementary Table S2.

List of predicted *C. parapsilosis* MFS proteins (Pfam clan CL0015) and their expression in cells grown in SD, S3OH and S4OH media (RNA-seq analysis).

Systematic Name	Gene Name	Domain acc.	Doman name	E-value	significance	SDS3OH	SDS4OH	S3OH basemean	S3OH log2foldchange	S3OH p value	S4OH basemean	S4OH log2foldchange	S4OH p value
CPAR2_100280		PF07690.14	MFS_1	9.50E-20	1	23.49	22.22	20.4985	-0.196808	0.8359	26.1745	0.236191	0.82215
CPAR2_100300	*TNA1*	PF07690.14	MFS_1	3.10E-28	1	188.29	178.09	223.998	0.250518	0.842	217.109	0.285806	0.84405
CPAR2_100330		PF07690.14	MFS_1	1.40E-22	1	8.30	7.85	61.4351	2.88708	0.008	30.5539	1.95973	0.0839
CPAR2_100460	*HBT4*	PF07690.14	MFS_1	3.40E-34	1	40.19	38.01	8.79128	-2.19266	0.0398	26.1118	-0.541753	0.6085
CPAR2_100470	*HBT3*	PF07690.14	MFS_1	5.60E-32	1	29.34	27.75	15.3002	-0.93939	0.33665	1337.84	5.59117	0.0126
CPAR2_100490		PF00083.22	Sugar_tr	8.10E-22	1	27.42	25.93	78.0574	1.50947	0.13755	127.411	2.29671	0.07615
CPAR2_101830		PF07690.14	MFS_1	2.00E-24	1	33.38	31.57	44.8152	0.425097	0.66775	59.1865	0.906731	0.4131
CPAR2_102100		PF07690.14	MFS_1	4.40E-27	1	21.54	20.37	13.0227	-0.725902	0.46565	15.1353	-0.428663	0.6848
CPAR2_102760		PF07690.14	MFS_1	1.10E-35	1	0.38	0.36	4.18931	3.45943	0.0504	4.29639	3.5762	0.0548
CPAR2_103530		PF07690.14	MFS_1	1.10E-22	1	49.35	46.68	19.9239	-1.30858	0.18925	16.9114	-1.46473	0.18385
CPAR2_107230		PF00083.22	Sugar_tr	2.50E-34	1	4.47	4.22	0.57593	-2.95525	0.07195	0.284778	-3.89095	0.0741
CPAR2_108210		PF00083.22	Sugar_tr	9.00E-100	1	11.35	10.74	17.0205	0.58457	0.5532	15.4995	0.52987	0.6194
CPAR2_108220		PF00083.22	Sugar_tr	1.60E-99	1	10.15	9.60	5.79514	-0.809097	0.4498	7.34518	-0.386789	0.72815
CPAR2_108340		PF00083.22	Sugar_tr	2.30E-99	1	0.70	0.66	0.443497	-0.65391	0.69105	24.9508	5.24047	0.0125
CPAR2_108350		PF00083.22	Sugar_tr	1.10E-97	1	4.18	3.95	0.89447	-2.22275	0.1185	1.27015	-1.6365	0.24315
CPAR2_108360		PF00083.22	Sugar_tr	8.30E-102	1	1.78	1.68	0.0685166	-4.69539	0.2171	0.0301149	-5.80101	0.2508
CPAR2_108370		PF00083.22	Sugar_tr	1.60E-100	1	7.57	7.16	49.8116	2.7185	0.01265	84.4722	3.56085	0.00685
CPAR2_108860		PF07690.14	MFS_1	1.10E-31	1	0.00	0.00	0	0	1	0	0	1
CPAR2_108880		PF07690.14	MFS_1	2.70E-28	1	59.86	56.62	34.7243	-0.785711	0.4249	33.3503	-0.763603	0.49495
CPAR2_109050		PF07690.14	MFS_1	1.10E-31	1	0.00	0.00	0	0	1	0	0	1
CPAR2_109920		PF00083.22	Sugar_tr	1.70E-83	1	9.18	8.68	15.4634	0.752715	0.4518	20.6924	1.25331	0.2533
CPAR2_200220		PF07690.14	MFS_1	7.20E-31	1	8.61	8.15	1.68219	-2.35631	0.0721	2.17283	-1.90672	0.14305
CPAR2_200970		PF07690.14	MFS_1	2.80E-46	1	4.98	4.71	0.522129	-3.25481	0.0501	1.13395	-2.05558	0.1405
CPAR2_201440		PF07690.14	MFS_1	1.30E-36	1	22.62	21.39	17.7473	-0.349962	0.71595	58.1093	1.44156	0.2008
CPAR2_202130		PF07690.14	MFS_1	2.70E-36	1	413.18	390.79	128.988	-1.67952	0.19265	121.879	-1.68095	0.2697
CPAR2_202420		PF07690.14	MFS_1	1.00E-26	1	23.20	21.94	7.22066	-1.68387	0.10355	26.2798	0.260238	0.8067
CPAR2_202480		PF00083.22	Sugar_tr	4.90E-89	1	23.38	22.11	43.8671	0.907966	0.3562	47.6392	1.10733	0.3251
CPAR2_203100		PF07690.14	MFS_1	1.00E-20	1	42.34	40.05	52.474	0.309573	0.7484	117.975	1.55873	0.2084
CPAR2_203280		PF07690.14	MFS_1	3.60E-20	1	11.34	10.72	5.62792	-1.0104	0.34505	5.31266	-1.01321	0.3744
CPAR2_203500		PF07690.14	MFS_1	2.70E-28	1	0.84	0.80	0.79895	-0.0754902	0.95905	4.43738	2.4784	0.11
CPAR2_203510		PF07690.14	MFS_1	7.00E-32	1	7.15	6.76	10.8094	0.597241	0.5787	49.7326	2.8795	0.01925
CPAR2_203870		PF07690.14	MFS_1	4.40E-41	1	0.16	0.15	0.0394773	-2.03242	1	0.0347026	-2.13805	1
CPAR2_203890		PF07690.14	MFS_1	2.20E-26	1	29.26	27.68	11.6132	-1.33318	0.70495	15.5496	-0.831714	0.85555
CPAR2_204840	*HBT2*	PF07690.14	MFS_1	8.00E-36	1	1.56	1.48	0.649138	-1.26689	0.4242	1847.59	10.2883	0.00215
CPAR2_207250		PF07690.14	MFS_1	2.20E-37	1	17.91	16.94	6.23054	-1.52372	0.1512	5.13271	-1.72301	0.14205
CPAR2_207260		PF07690.14	MFS_1	3.60E-38	1	6.46	6.11	4.05875	-0.669851	0.5685	3.97472	-0.619681	0.61095
CPAR2_207270		PF07690.14	MFS_1	3.90E-40	1	50.30	47.57	13.4224	-1.90585	0.0652	21.0316	-1.17757	0.2812
CPAR2_207540		PF07690.14	MFS_1	9.20E-36	1	16.25	15.37	2.74967	-2.56294	0.037	5.30652	-1.53408	0.1792
CPAR2_208110		PF07690.14	MFS_1	7.10E-23	1	88.42	83.63	37.3658	-1.24269	0.22195	13.9681	-2.58192	0.03145
CPAR2_208160		PF00083.22	Sugar_tr	9.00E-39	1	0.00	0.00	0	0	1	0	0	1
CPAR2_208190		PF00083.22	Sugar_tr	9.00E-39	1	0.05	0.04	0	-inf	1	0	-inf	1
CPAR2_209010		PF07690.14	MFS_1	9.90E-21	1	27.90	26.39	67.6698	1.2783	0.1954	61.2247	1.21426	0.2736
CPAR2_209680		PF07690.14	MFS_1	2.10E-41	1	5.87	5.56	2.88385	-1.02647	0.39305	14.115	1.34504	0.23495
CPAR2_209690		PF07690.14	MFS_1	5.70E-37	1	19.91	18.83	11.4642	-0.796022	0.423	17.7454	-0.0853584	0.9349
CPAR2_209700		PF07690.14	MFS_1	3.30E-41	1	31.89	30.16	13.7672	-1.21189	0.2247	15.5599	-0.954945	0.377
CPAR2_210080		PF00083.22	Sugar_tr	2.20E-114	1	106.89	101.10	100.562	-0.0879802	0.93445	81.2022	-0.316119	0.80485
CPAR2_210100		PF03239.12	FTR1	7.70E-79	1	111.46	105.42	5.85141	-4.25154	0.00095	6.16428	-4.09604	0.0033
CPAR2_210150		PF00083.22	Sugar_tr	1.60E-149	1	7.97	7.54	10.3592	0.377454	0.71905	14.6288	0.955708	0.3937
CPAR2_210750		PF07690.14	MFS_1	4.40E-18	1	111.75	105.69	18.6807	-2.58061	0.01735	21.4112	-2.30344	0.06345
CPAR2_212850		PF00083.22	Sugar_tr	2.00E-148	1	1.14	1.08	0.0322931	-5.1479	0.25115	0.113549	-3.25352	0.1846
CPAR2_212860		PF00083.22	Sugar_tr	3.10E-149	1	9543.07	9026.08	10.8938	-9.7748	0.00325	4.9633	-10.8286	0.00335
CPAR2_300590		PF07690.14	MFS_1	4.50E-38	1	11.90	11.26	67.7745	2.50925	0.01715	69.2217	2.62009	0.0311

CPAR2_300670		PF07690.14	MFS_1	2.40E-27	1	3.16	2.98	0.795411	-1.98803	0.18625	0.730991	-2.02952	0.19955
CPAR2_300680		PF07690.14	MFS_1	1.20E-39	1	4.75	4.49	1.94448	-1.28768	0.30265	7.05775	0.652501	0.58495
CPAR2_300730		PF07690.14	MFS_1	2.80E-36	1	13.60	12.86	15.7986	0.216718	0.82665	1.53998	-3.06174	0.02625
CPAR2_300740		PF07690.14	MFS_1	1.80E-35	1	13.89	13.13	2.27577	-2.60914	0.034	0.512955	-4.67824	0.0181
CPAR2_300750		PF07690.14	MFS_1	1.80E-38	1	25.10	23.74	8.71913	-1.52546	0.13915	6.10328	-1.95971	0.0872
CPAR2_300760		PF07690.14	MFS_1	5.60E-37	1	28.53	26.98	135.44	2.24733	0.0474	89.7356	1.73378	0.1578
CPAR2_300770		PF07690.14	MFS_1	2.20E-37	1	4.70	4.45	5.26191	0.163039	0.8857	15.256	1.77911	0.1265
CPAR2_301390		PF00083.22	Sugar_tr	5.40E-101	1	3.23	3.06	100.251	4.95389	0.00025	29.3946	3.26426	0.0149
CPAR2_301470		PF07690.14	MFS_1	4.10E-15	1	25.00	23.64	43.0502	0.784167	0.424	53.7172	1.18388	0.2827
CPAR2_301480		PF00083.22	Sugar_tr	1.70E-17	1	8.26	7.81	9.1925	0.154782	0.8813	12.1531	0.637931	0.56945
CPAR2_301640		PF07690.14	MFS_1	3.90E-38	1	1.41	1.34	211.683	7.22536	0.0015	81.1854	5.9231	0.00055
CPAR2_301750		PF07690.14	MFS_1	1.30E-38	1	0.74	0.70	8.21205	3.47454	0.0231	4.91282	2.8137	0.065
CPAR2_301760	*MDR1*	PF07690.14	MFS_1	1.30E-34	1	23.64	22.36	7.04113	-1.74744	0.0944	80.1426	1.8416	0.11665
CPAR2_302090		PF06813.11	Nodulin-like	4.00E-18	1	25.60	24.21	46.3653	0.856894	0.3772	41.2399	0.768242	0.47725
CPAR2_400100		PF07690.14	MFS_1	2.20E-31	1	74.48	70.44	37.134	-1.00402	0.3186	45.9753	-0.615547	0.5915
CPAR2_400160		PF07690.14	MFS_1	1.30E-31	1	3.49	3.30	14.0331	2.00774	0.0956	12.6987	1.94393	0.1215
CPAR2_400230		PF07690.14	MFS_1	1.00E-31	1	18.70	17.68	23.6601	0.339647	0.7213	43.9487	1.31337	0.226
CPAR2_400790		PF00083.22	Sugar_tr	4.10E-87	1	12.76	12.07	8.48295	-0.589312	0.56425	9.1905	-0.393381	0.71545
CPAR2_401450		PF00083.22	Sugar_tr	4.00E-76	1	1.51	1.43	348.366	7.84881	0.0004	270.213	7.56266	0.0012
CPAR2_402040		PF00083.22	Sugar_tr	4.10E-45	1	6.75	6.38	857.364	6.98889	0.0008	932.711	7.19076	0.0025
CPAR2_402050		PF00083.22	Sugar_tr	2.10E-38	1	26.30	24.88	36.0242	0.453869	0.6448	40.4443	0.701193	0.51545
CPAR2_402410		PF01733.16	Nucleoside_tran	1.40E-56	1	29.77	28.16	22.3291	-0.415098	0.66965	35.52	0.334958	0.7464
CPAR2_402960		PF13000.5	Acatn	3.80E-121	1	37.61	35.58	37.2736	-0.0131268	0.98835	34.012	-0.0648841	0.95295
CPAR2_403640		PF07690.14	MFS_1	4.90E-42	1	92.58	87.57	55.8122	-0.730182	0.478	80.4031	-0.123157	0.9218
CPAR2_403890		PF07690.14	MFS_1	3.40E-33	1	8.22	7.78	11.3822	0.469074	0.6547	8.65686	0.154562	0.8931
CPAR2_405530		PF00083.22	Sugar_tr	1.80E-121	1	11.35	10.73	21.907	0.949122	0.3309	41.1804	1.94004	0.0914
CPAR2_407080		PF07690.14	MFS_1	2.10E-18	1	232.80	220.19	57.6271	-2.01429	0.0905	55.0096	-2.001	0.143
CPAR2_407240		PF07690.14	MFS_1	6.20E-24	1	21.99	20.80	62.1412	1.49843	0.13765	23.3708	0.167941	0.87245
CPAR2_407250		PF07690.14	MFS_1	1.60E-31	1	13.43	12.70	11.9888	-0.163666	0.87025	9.84874	-0.366987	0.7315
CPAR2_407290		PF07690.14	MFS_1	2.10E-33	1	29.39	27.80	8.81319	-1.7375	0.0935	16.3414	-0.766348	0.46975
CPAR2_407300		PF07690.14	MFS_1	7.70E-32	1	36.25	34.28	21.5156	-0.752541	0.4464	25.2805	-0.439544	0.67775
CPAR2_407310		PF07690.14	MFS_1	3.00E-34	1	26.70	25.26	28.5181	0.0949116	0.92355	957.031	5.24388	0.0109
CPAR2_407560		PF07690.14	MFS_1	6.60E-11	1	1.11	1.05	1.44702	0.382616	0.7864	1.21901	0.215591	0.8866
CPAR2_407570		PF07690.14	MFS_1	2.90E-08	1	2.44	2.31	0.121257	-4.3301	0.14435	0.106591	-4.43573	0.1534
CPAR2_407640		PF00083.22	Sugar_tr	1.40E-52	1	5.72	5.41	7.0696	0.306523	0.78325	22.2974	2.04405	0.08295
CPAR2_501920		PF07690.14	MFS_1	8.80E-35	1	14.68	13.89	19.7525	0.428076	0.6559	31.8288	1.19673	0.27835
CPAR2_502820		PF07690.14	MFS_1	2.10E-34	1	99.55	94.15	8.35015	-3.5755	0.0021	6.89918	-3.77053	0.0062
CPAR2_503540	*PTR2*	PF00854.19	PTR2	4.00E-93	1	71.60	67.72	98.1576	0.455205	0.66485	126.711	0.903923	0.4905
CPAR2_503880		PF07690.14	MFS_1	1.10E-14	1	8.22	7.78	9.04266	0.137504	0.8946	55.7705	2.84254	0.01855
CPAR2_504080		PF00083.22	Sugar_tr	4.30E-88	1	2.21	2.09	33.8569	3.93786	0.0043	50.1585	4.58525	0.0024
CPAR2_504220		PF07690.14	MFS_1	2.90E-36	1	24.57	23.24	17.1447	-0.519309	0.5958	24.9475	0.102176	0.92295
CPAR2_600450	*HGT10*	PF00083.22	Sugar_tr	1.20E-113	1	5.83	5.51	9.3607	0.684025	0.5252	11.4397	1.05374	0.3489
CPAR2_600460		PF00083.22	Sugar_tr	1.50E-115	1	6.36	6.02	53.6202	3.07566	0.0074	208.81	5.11736	0.0038
CPAR2_601960		PF07690.14	MFS_1	1.50E-21	1	30.42	28.77	8.58954	-1.8245	0.08025	8.28568	-1.7961	0.1126
CPAR2_602760		PF07690.14	MFS_1	5.40E-36	1	9.52	9.00	24.8845	1.38629	0.16745	39.8672	2.1466	0.06245
CPAR2_603010		PF07690.14	MFS_1	1.10E-35	1	1.96	1.85	7.23824	1.8867	0.12755	137.646	6.21623	0.00085
CPAR2_700010		PF07690.14	MFS_1	1.40E-21	1	2.97	2.81	31.1218	3.38847	0.0108	72.0344	4.67958	0.003
CPAR2_700110	*NAG4*	PF07690.14	MFS_1	4.10E-40	1	11.12	10.52	87.776	2.98075	0.0057	168.628	4.00305	0.00755
CPAR2_700120		PF07690.14	MFS_1	1.10E-38	1	0.41	0.39	0.547691	0.400538	1	11.5013	4.87318	0.0279
CPAR2_700570	*FTR1*	PF03239.12	FTR1	2.20E-96	1	152.60	144.33	7.46358	-4.3537	0.00115	6.20383	-4.54005	0.00255
CPAR2_701650		PF00083.22	Sugar_tr	9.40E-105	1	28.19	26.67	19.6393	-0.52169	0.5944	29.2521	0.13346	0.89675
CPAR2_702940		PF07690.14	MFS_1	2.00E-08	1	6.06	5.73	9.51274	0.651359	0.53825	8.53702	0.575584	0.6181
CPAR2_702950		PF07690.14	MFS_1	2.90E-12	1	0.77	0.73	1.32851	0.787006	0.60145	0.49633	-0.553083	0.7441
CPAR2_702960		PF07690.14	MFS_1	1.90E-15	1	1.01	0.96	1.18168	0.222392	0.88015	3.38339	1.82038	0.21265
CPAR2_702970		PF07690.14	MFS_1	8.20E-13	1	0.09	0.09	0.475624	2.3599	1	1.13484	3.69484	0.2041

CPAR2_703800		PF00083.22	Sugar_tr	3.10E-91	1	3.03	2.87	1.42337	-1.09132	0.4113	1.43369	-1.00054	0.4613
CPAR2_704330	*HBT1*	PF07690.14	MFS_1	2.10E-34	1	1.08	1.02	1514.63	10.4526	0.00305	3.58325	1.80949	0.21825
CPAR2_800280		PF07690.14	MFS_1	2.00E-26	1	28.13	26.61	36.8963	0.391289	0.6817	31.5254	0.244681	0.81135
CPAR2_800760		PF00854.19	PTR2	4.50E-55	1	22.82	21.59	3.36466	-2.76203	0.0196	6.36661	-1.76162	0.1182
CPAR2_801480		PF07690.14	MFS_1	2.50E-20	1	148.71	140.66	43.8272	-1.76262	0.1077	34.9502	-2.00879	0.1182
CPAR2_801490		PF07690.14	MFS_1	1.10E-17	1	49.18	46.51	27.0896	-0.860266	0.3798	27.4632	-0.760152	0.48685
CPAR2_801500		PF07690.14	MFS_1	1.60E-08	1	12.92	12.22	14.1809	0.134228	0.89355	12.4658	0.0286042	0.97885
CPAR2_801660		PF00083.22	Sugar_tr	4.60E-26	1	0.28	0.27	0.259513	-0.125531	1	0.136875	-0.968121	1
CPAR2_802070		PF00083.22	Sugar_tr	7.80E-82	1	1.35	1.28	1.31105	-0.0441033	0.9758	1.68656	0.399611	0.7819
CPAR2_802240		PF07690.14	MFS_1	6.10E-34	1	373.35	353.13	26.4363	-3.81994	0.00735	10.5957	-5.05864	0.00495
CPAR2_802600		PF00083.22	Sugar_tr	1.10E-98	1	66.75	63.14	33.2925	-1.00366	0.31155	50.3653	-0.326069	0.77345
CPAR2_802690		PF07690.14	MFS_1	6.30E-37	1	2.86	2.70	0.684613	-2.06217	0.1956	0.752264	-1.84586	0.2515
CPAR2_802700		PF07690.14	MFS_1	2.10E-35	1	5.70	5.39	8.63309	0.599084	0.5846	17.2558	1.67857	0.14215
CPAR2_802710		PF07690.14	MFS_1	1.50E-36	1	8.75	8.28	9.36769	0.0979752	0.9254	17.9943	1.12011	0.3093
CPAR2_802720		PF07690.14	MFS_1	6.90E-39	1	0.10	0.09	0.676459	2.80047	0.26725	126.596	10.4288	0.18885
CPAR2_802890		PF07690.14	MFS_1	1.10E-31	1	67.99	64.30	19.482	-1.80314	0.0795	23.9995	-1.42192	0.2112
CPAR2_803030		PF07690.14	MFS_1	1.70E-10	1	12.59	11.91	1.8528	-2.76501	0.03175	1.18452	-3.33006	0.02015
CPAR2_803120		PF03239.12	FTR1	9.90E-23	1	22.58	21.36	40.0458	0.826464	0.40265	31.4591	0.558642	0.6051
CPAR2_804220		PF00854.19	PTR2	1.30E-51	1	2.40	2.27	1.07976	-1.15373	0.4063	1.74847	-0.377996	0.78435
CPAR2_804280		PF07690.14	MFS_1	3.00E-14	1	9.29	8.79	19.2293	1.04952	0.29535	13.0925	0.575307	0.5883
CPAR2_804310	*TPO3*	PF07690.14	MFS_1	6.70E-34	1	417.84	395.20	23.9253	-4.12633	0.0076	262.464	-0.590468	0.72225
CPAR2_804410		PF07690.14	MFS_1	1.30E-09	1	31.42	29.72	11.967	-1.39274	0.1749	19.337	-0.620082	0.55675
CPAR2_804430		PF00854.19	PTR2	3.40E-52	1	2.75	2.60	1.30708	-1.07364	0.42595	2.27301	-0.195036	0.88
CPAR2_804630		PF07690.14	MFS_1	1.60E-34	1	5.98	5.65	34.6411	2.53478	0.01975	52.8183	3.22368	0.0108
CPAR2_805630		PF03239.12	FTR1	1.80E-94	1	478.95	453.01	53.1777	-3.17099	0.0195	39.4763	-3.52047	0.0256
CPAR2_807300		PF00083.22	Sugar_tr	5.60E-99	1	21.85	20.67	54.9177	1.32947	0.1929	40.3859	0.9664	0.3806
CPAR2_807810		PF07690.14	MFS_1	3.90E-11	1	152.40	144.14	38.6715	-1.97852	0.0638	32.402	-2.15335	0.0849
CPAR2_808330		PF07690.14	MFS_1	7.10E-36	1	9.28	8.78	3.01312	-1.62303	0.1728	16.1415	0.878763	0.41995
CPAR2_808390		PF07690.14	MFS_1	8.00E-33	1	9.91	9.37	0.976186	-3.34362	0.02715	28.7311	1.61604	0.1494
CPAR2_808400		PF07690.14	MFS_1	3.50E-32	1	0.46	0.43	0.534669	0.230613	1	29.4339	6.09366	0.0258
CPAR2_808410		PF07690.14	MFS_1	1.10E-32	1	0.35	0.33	50.4363	7.19124	0.0455	223.715	9.42072	0.0484

Supplementary Table S3. Several amino acid residues conserved between the bacterial AAHS and *C. parapsilosis* Hbt proteins are important for the transport activity of AAHS permeases.

MFS carrier	Uniprot ID	Amino acid residues*														
**Bacterial AAHS transporters**																
GenK (*C. glutamicum*)	Q8NLB7	D54A<br>D54E	D57A<br>D57E	G98	D102	R103A	G105	R137	D157	R199	W309V	D312A	R313A	I317H<br>I317Y	R374	R386A
MhbT (*K. pneumoniae*)	Q5EXK5	D34	D37	G78	D82A	R83	G85	R117	E137	H176	V311W	D314A	R315	Y319	R377	R389
MhpT (*E. coli*)	P77589	E27A<br>E27D	D30	G71	D75A<br>D75E	R76	G78	R110	E130	Q168	A272H	D275	K276D	V280	R337	R349
PcaK (*P. putida*)	Q51955	D41A<br>D41N<br>D41E	D44A<br>D44N<br>D44E	G85V	D89N	R90	G92L<br>G92V<br>G92A<br>G92Q<br>G92C	R124A	E144A	H183A	W320	D323N	R324	H328A<br>H328R	R386A	R398A
**Consensus**		**D / E**	**D**	**G**	**D**	**R**	**G**	**R**	**E / D**			**D**	**R / K**		**R**	**R**
***C. parapsilosis* Hbt proteins**																
Hbt1 (CPAR2_704330)	G8BL03	S82	M85	V126	N130	A131	G133	R165	D185	K224	P349	D352	W353	R366	K434	R446
Hbt2 (CPAR2_204840)	G8BG57	G83	M86	V127	Q131	R132	G134	R166	E186	K225	Y351	D354	R355	S368	K431	R443
Hbt3 (CPAR2_100470)	G8B6C7	T94	L97	V138	D142	R143	G145	R177	E197	K236	Y362	D365	R366	S379	K441	R454
Hbt4 (CPAR2_100460)	G8B6C6	A77	M80	V121	E125	R126	G128	R160	E180	K219	Y345	D348	K349	S362	K424	R437
**Consensus**			**M / L**	**V**			**G**	**R**	**E / D**	**K**		**D**			**K**	**R**

* - Mutations that abolish or substantially decrease the transport activity are shown in red. Transporters carrying mutations shown in blue retained the transport activity above 40 % of the wild type permease and those shown in violet exhibit increased substrate transport[1-6].

Supplementary Table S4.
Expression of the genes *MNX1-2 and HBT1-4* in *C. parapsilosis* cells analyzed by qPCR.

	Mean	SD	SEM	Fold change to SD medium
MNX1				
**SD**	0.002445	0.000985	0.000569	1.00
**SGly**	0.009024	0.010249	0.005917	3.69
**SEtOH**	0.022934	0.005802	0.003350	9.38
**S3OH**	0.038241	0.005169	0.002984	15.64
**S2,5diOH**	0.634234	0.080831	0.046668	259.42
**S4OH**	3.039887	2.044622	1.180463	1243.40
**S2,4diOH**	2.166942	1.057166	0.610355	886.34
**S3,4diOH**	2.691400	0.751894	0.434106	1100.86
**SHyd**	0.162359	0.093223	0.053822	66.41
**SRes**	0.275936	0.271816	0.156933	112.87
MNX2				
**SD**	0.001746	0.000949	0.000548	1.00
**SGly**	0.002438	0.001807	0.001043	1.40
**SEtOH**	0.002348	0.001547	0.000893	1.34
**S3OH**	1.543663	1.400050	0.808319	884.34
**S2,5diOH**	0.419579	0.275436	0.159023	240.37
**S4OH**	0.052480	0.075789	0.043757	30.07
**S2,4diOH**	0.000391	0.000047	0.000027	0.22
**S3,4diOH**	0.017151	0.008066	0.004657	9.83
**SHyd**	0.130973	0.198485	0.114596	75.03
**SRes**	0.252837	0.428213	0.247229	3.21
HBT1				
**SD**	0.000418	0.000252	0.000146	1.00
**SGly**	0.002269	0.000404	0.000233	5.43
**SEtOH**	0.005026	0.005390	0.003112	12.03
**S3OH**	0.352166	0.250453	0.144599	843.01
**S2,5diOH**	0.466514	0.296230	0.171029	1116.74
**S4OH**	0.012304	0.016260	0.009388	29.45
**S2,4diOH**	0.000721	0.000490	0.000283	1.72
**S3,4diOH**	0.023128	0.002278	0.001315	55.36
**SHyd**	0.328057	0.475389	0.274466	785.30
**SRes**	0.010035	0.001064	0.000614	24.02
HBT2				
**SD**	0.000983	0.000370	0.000214	1.00
**SGly**	0.005308	0.002496	0.001441	5.40
**SEtOH**	0.005096	0.004308	0.002487	5.18
**S3OH**	0.000232	0.000355	0.000205	0.24
**S2,5diOH**	0.461098	0.190840	0.110182	468.85
**S4OH**	0.116142	0.098361	0.056789	118.09
**S2,4diOH**	0.060128	0.072149	0.041655	61.14
**S3,4diOH**	0.056442	0.014821	0.008557	57.39
**SHyd**	0.407528	0.492900	0.284576	414.38
**SRes**	0.168631	0.103752	0.059901	171.46

HBT3				
**SD**	0.080996	0.060965	0.035198	1.00
**SGly**	0.141925	0.061683	0.035613	1.75
**SEtOH**	0.063983	0.018632	0.010757	0.79
**S3OH**	0.062378	0.067171	0.038781	0.77
**S2,5diOH**	0.502738	0.017986	0.010384	6.21
**S4OH**	0.652068	0.338806	0.195609	8.05
**S2,4diOH**	0.085714	0.066216	0.038230	1.06
**S3,4diOH**	0.236075	0.031278	0.018058	2.91
**SHyd**	0.412536	0.410344	0.236912	5.09
**SRes**	0.202240	0.000954	0.000551	2.50

HBT4				
**SD**	0.051206	0.032281	0.018638	1.00
**SGly**	0.075692	0.044197	0.025517	1.48
**SEtOH**	0.046257	0.013578	0.007839	0.90
**S3OH**	0.019360	0.019821	0.011443	0.38
**S2,5diOH**	0.523618	0.168826	0.097472	10.23
**S4OH**	0.049894	0.045684	0.026376	0.97
**S2,4diOH**	0.058112	0.067951	0.039232	0.37
**S3,4diOH**	0.047686	0.004829	0.002788	0.93
**SHyd**	0.597703	0.495363	0.285998	11.67
**SRes**	0.446520	0.067370	0.038896	8.72

Supplementary Table S5.
Phenotypic characterization of *C. parapsilosis Δhbt* mutants.

**Assay**	**Colony morphology**	**Pseudohyphal growth**	**Growth kinetics**	**Sensitivity to stress**								
**Medium**	**$SD_{1\%}$**	**DMEM + FBS**	**YPD**	**YPD**	**YPD**	**YPD**	**YPD**	**YPD**	**YPD**	**YPD**	**YPD**	**YPD**
				**pH 4-8**	**Caclofluor white**	**Congo red**	**Caffeine**	**hydrogen peroxide**	**SDS**	**Hygromycin B**	**Caspofungin**	**Fluconazole**
**Temperature**	**30°C**	**37°C**	**30°C**	**30°C**	**30°C**	**30°C**	**30°C**	**30°C**	**30°C**	**30°C**	**30°C**	**30°C**
wild-type	Rough	Normal	Normal	Normal	Normal	Normal	Normal	Normal	Normal	Normal	Normal	Normal
Δhbt1/Δhbt1	Rough	Normal	Normal	Normal	Normal	Normal	Normal	Normal	Normal	Normal	Normal	Normal
Δhbt2/Δhbt2	Rough	Normal	Normal	Normal	Normal	Normal	Normal	Normal	Normal	Normal	Normal	Normal
Δhbt3/Δhbt3	Rough	Normal	Normal	Normal	Normal	Normal	Normal	Normal	Normal	Normal	Normal	Normal
Δhbt4/Δhbt4	Rough	Normal	Normal	Normal	Normal	Normal	Resistant	Normal	Normal	Normal	Sensitive	Resistant

**Assay**	**Temperature sensitivity**											
**Medium**	**YPD**			**YCB + BSA**			**$SD_{1\%}$**			**$SD_{1\%}$ + FBS**		
**Temperature**	**20°C**	**30°C**	**37°C**	**20°C**	**30°C**	**37°C**	**20°C**	**30°C**	**37°C**	**20°C**	**30°C**	**37°C**
wild-type	Normal	Normal	Normal	Normal	Normal	Normal	Normal	Normal	Normal	Normal	Normal	Normal
Δhbt1/Δhbt1	Normal	Normal	Normal	Normal	Normal	Normal	Normal	Normal	Normal	Normal	Normal	Normal
Δhbt2/Δhbt2	Normal	Normal	Normal	Normal	Normal	Normal	Normal	Normal	Normal	Normal	Normal	Normal
Δhbt3/Δhbt3	Normal	Normal	Normal	Normal	Normal	Normal	Normal	Normal	Normal	Normal	Normal	Normal
Δhbt4/Δhbt4	Normal	Normal	Normal	Slow	Normal	Normal	Slow	Normal	Normal	Slow	Normal	Normal

Supplementary Table S6.
The list of oligonucleotides used in this study.

Oligo	*Gene*	ORF	Sequence [5' -> 3']
**Transcript quantification by real-time qPCR analysis**			
MNX1-F	*MNX1*	CPAR2_102790	AGAGCTTGGAAGGAGTTTAAGG
MNX1-R	*MNX1*	CPAR2_102790	GACACATCCTTCTCACCACTAC
MNX2-F	*MNX2*	CPAR2_704320	GGTGTTGAAATGGGTGTTGGCTGA
MNX2-R	*MNX2*	CPAR2_704320	AGCACCTTGAGCCAAGTACGGTAA
HBT1-F	*HBT1*	CPAR2_704330	GCCATTCTTGACTGTGGCTGCTTT
HBT1-R	*HBT1*	CPAR2_704330	ACGCTAAGGACCACCAACCATTCT
HBT2-F	*HBT2*	CPAR2_204840	CAATGAGATGTTTGCTGGCGGTGT
HBT2-R	*HBT2*	CPAR2_204840	GGAAAGCACCAAACATGGTTCCCA
HBT3-F	*HBT3*	CPAR2_100470	ATCGGTACAGCCATCGGTGAAGTT
HBT3-R	*HBT3*	CPAR2_100470	AAAGAAGCAAACGCACACCCAACC
HBT4-F	*HBT4*	CPAR2_100460	TGGGAGCTTTGTTGATGGGCATTG
HBT4-R	*HBT4*	CPAR2_100460	GGCCCTAAACCGCAACTGTTTGAA
**Cloning ORFs into the pPK5 plasmid vector**			
HBT1_FWD	*HBT1*	CPAR2_704330	CATGGGGATCCTCTAGAGTGGCCATTCATTGCAAGG
HBT1_REV	*HBT1*	CPAR2_704330	CTTAGACATGTCGAGGACTGCTTTGATGGCACGTCTAG
HBT2_FWD	*HBT2*	CPAR2_204840	ACCATGGGGATCCTCTAGAGGATGAAACTCCTGCCAAGACAG
HBT2_REV	*HBT2*	CPAR2_204840	CACCTTTAGACATGTCGAGGGCTCTGCCATGCCTCTGCTCCCAA
HBT3_FWD	*HBT3*	CPAR2_100470	ACCATGGGGATCCTCTAGAGATTGATCTATCTGCATTCGAGA
HBT3_REV	*HBT3*	CPAR2_100470	CACCTTTAGACATGTCGAGGGCCTTTCCTTTACTTGCAGCTAAA
**Construction of the knockout strains**			
**UpFw primer 1**			
HBT1_UpFw primer 1	*HBT1*	CPAR2_704330	GGAGGGGGGGTTCTCATAAG
HBT2_UpFw primer 1	*HBT2*	CPAR2_204840	TCCACTAAAAAGTTTAATTT
HBT3_UpFw primer 1	*HBT3*	CPAR2_100470	GTAACATTCACTAAATCTAC
HBT4_UpFw primer 1	*HBT4*	CPAR2_100460	GCTTCTTGGCCTTGTAATCC
**UpRev primer 3**			
HBT1_UpRev primer 3	*HBT1*	CPAR2_704330	cacggcgcgcctagcagcggAGTTAGTTGTGTTTGGTTGA
HBT2_UpRev primer 3	*HBT2*	CPAR2_204840	cacggcgcgcctagcagcggTGAATGTTGAGTTGATAAAA
HBT3_UpRev primer 3	*HBT3*	CPAR2_100470	cacggcgcgcctagcagcggTACTATTTATATCAGCTAAT
HBT4_UpRev primer 3	*HBT4*	CPAR2_100460	cacggcgcgcctagcagcggCGCTACTTGTGATACTGTCA
**DownFw primer 4**			
HBT1_DownFw primer 4	*HBT1*	CPAR2_704330	gtcagcggccgcatccctgcGTGTCTAGATTTATAGTATA
HBT2_DownFw primer 4	*HBT2*	CPAR2_204840	gtcagcggccgcatccctgcAATTTGTATAGTGAAATTTG
HBT3_DownFw primer 4	*HBT3*	CPAR2_100470	gtcagcggccgcatccctgcCAGTACTGCTTGAAAGAGTG
HBT4_DownFw primer 4	*HBT4*	CPAR2_100460	gtcagcggccgcatccctgcCAAGTGAGAGGAAAGGTTGT
**DownRev primer 6**			
HBT1_DownRev primer 6	*HBT1*	CPAR2_704330	GCTATCGAGGAAGACAATGG
HBT2_DownRev primer 6	*HBT2*	CPAR2_204840	CGACATATCGCAAAACGATG
HBT3_DownRev primer 6	*HBT3*	CPAR2_100470	GAAGACTCATTCAGTATGTT
HBT4_DownRev primer 6	*HBT4*	CPAR2_100460	ATATCTCGAGGCGATGTAAT
**Primer 2**			
Univ_Primer_2	*HBT1*<br>*HBT2*<br>*HBT3*<br>*HBT4*	CPAR2_704330<br>CPAR2_204840<br>CPAR2_100470<br>CPAR2_100460	ccgctgctaggcgcgccgtgACCAGTGTGATGGATATCTGC
**Primer 5 carrying TAG**			
HBT1_Primer_5	*HBT1*	CPAR2_704330	gcagggatgcggccgctgacGGGCCAGCAATAAAGCTCCAagctcggatccactagtaacg
HBT2_Primer_5	*HBT2*	CPAR2_204840	gcagggatgcggccgctgacCTGTCTCCAGTAGGTCAGATagctcggatccactagtaacg
HBT3_Primer_5	*HBT3*	CPAR2_100470	gcagggatgcggccgctgacACAGAGCTACAAGGTTTGGAagctcggatccactagtaacg
HBT4_Primer_5	*HBT4*	CPAR2_100460	gcagggatgcggccgctgacACAGACTCCCTAAGATGCAGagctcggatccactagtaacg
**Upstream check primer**			
HBT1_upstream_check_primer	*HBT1*	CPAR2_704330	GCCATTCATTGCAAGGAGGA
HBT2_upstream_check_primer	*HBT2*	CPAR2_204840	AGATGATGAAACTCCTGCCA
HBT3_upstream_check_primer	*HBT3*	CPAR2_100470	TTCCCCAATTGATCTATCTG
HBT4_upstream_check_primer	*HBT4*	CPAR2_100460	CTACCCAGCCCCACAAATAA
**Downstream check primer**			
HBT1_downstream_check_primer	*HBT1*	CPAR2_704330	TGCTGAATCTAACATCAAGT
HBT2_downstream_check_primer	*HBT2*	CPAR2_204840	GTTGCTCATACTTTTTCAGG
HBT3_downstream_check_primer	*HBT3*	CPAR2_100470	TTTGTGTCTAACATCTCCAT
HBT4_downstream_check_primer	*HBT4*	CPAR2_100460	CTACCCAGCCCCACAAATAA
**Primers for verification by qPCR**			
HBT1_Fw_qPCR	*HBT1*	CPAR2_704330	TCTGCTGGTTTAATGTGTGGTC
HBT1_Rev_qPCR	*HBT1*	CPAR2_704330	TTGATGGCACGTCTAGCACC
HBT2_Fw_qPCR	*HBT2*	CPAR2_204840	GGTTACTTGTCGGACAGATTGG
HBT2_Rev_qPCR	*HBT2*	CPAR2_204840	TAGCCTGGATGGGTAACCC
HBT3_Fw_qPCR	*HBT3*	CPAR2_100470	TTTGCTTTAGGTCCGCAAGC
HBT3_Rev_qPCR	*HBT3*	CPAR2_100470	CCCAAGCAACTAACCGATCAG
HBT4_Fw_qPCR	*HBT4*	CPAR2_100460	ATTGGGAAGTGTGTTTAGTACCAC
HBT4_Rev_qPCR	*HBT4*	CPAR2_100460	CCACCGACTGCTAATCCAGG

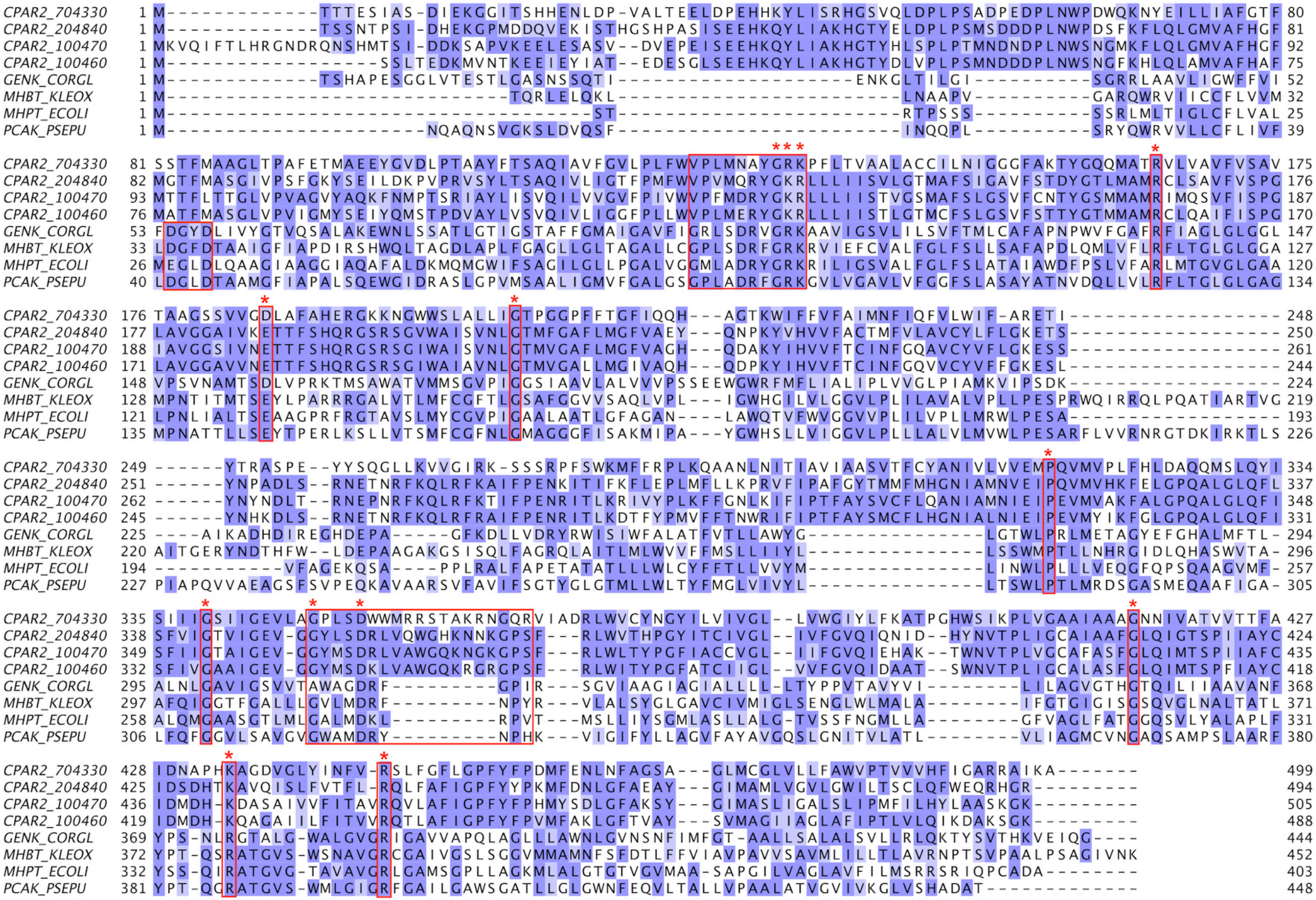

Supplementary Figure S1. Amino acid sequence alignment of the *C. parapsilosis* Hbt transporters and bacterial AAHS carriers GenK, MhbT, MhpT and PcaK. Conserved sequence motifs found in bacterial AAHS proteins (‘DGXD’ and ‘GXXXD(R/K)XGR(R/K)’) and the yeast Hbt transporters (‘VPXMX(R/A)YG(K/R)(R/K)’ and ‘G(Y/P)(M/L)SDX(L/W)(V/M)X(W/R)’) are shown. Note that the yeast motifs are similar to the consensus ‘GXXXD(R/K)XGR(R/K)’ of bacterial permeases. Asterisks indicate amino acid residues shared by bacterial and yeast transporters.

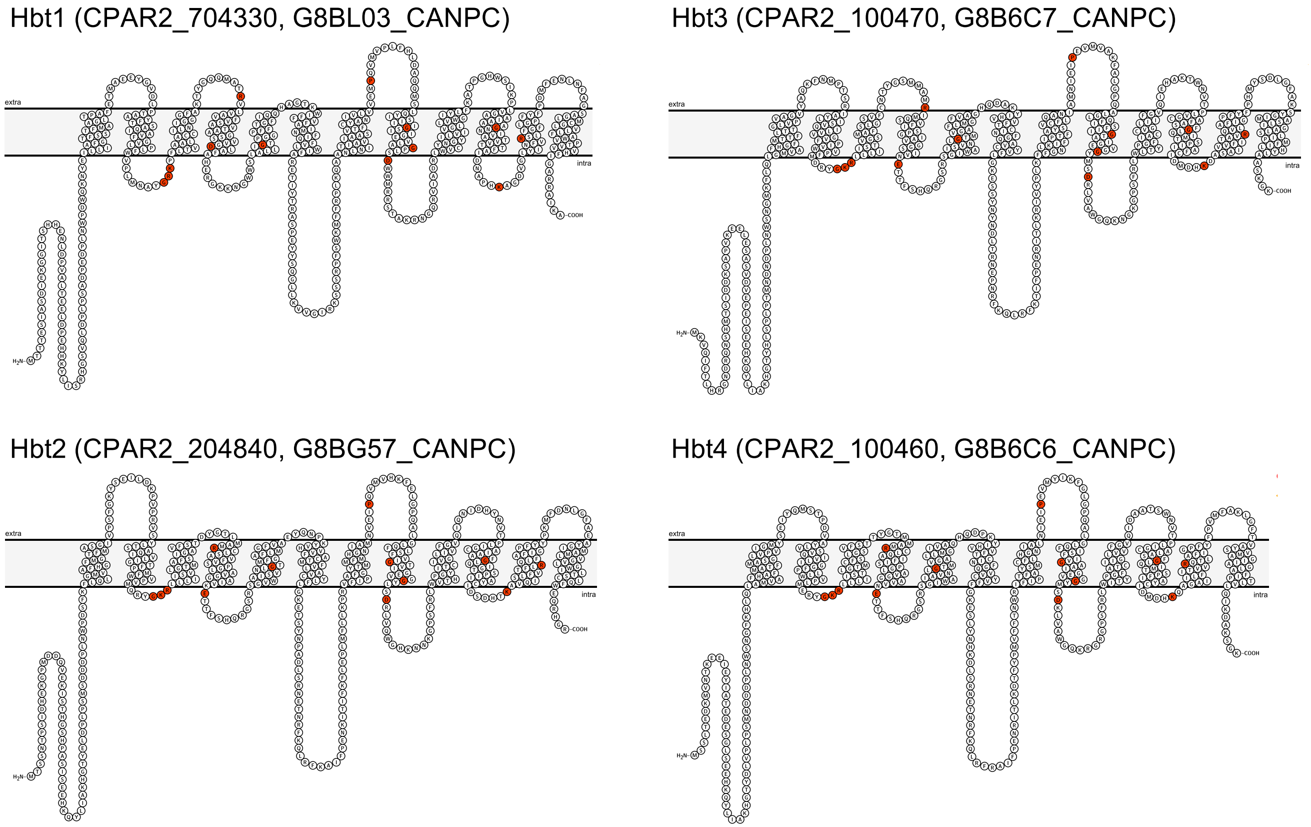

Supplementary Figure S2. *C. parapsilosis* MFS transporters Hbt1-4 have 12 predicted TMHs. Predictions of the protein topologies were inferred from the entries in the Uniprot database (http://www.uniprot.org/; accession numbers are shown in parentheses) and visualized using Protter (http://wlab.ethz.ch/protter/). Amino acid residues shared with bacterial AAHS transporters are shown in red.

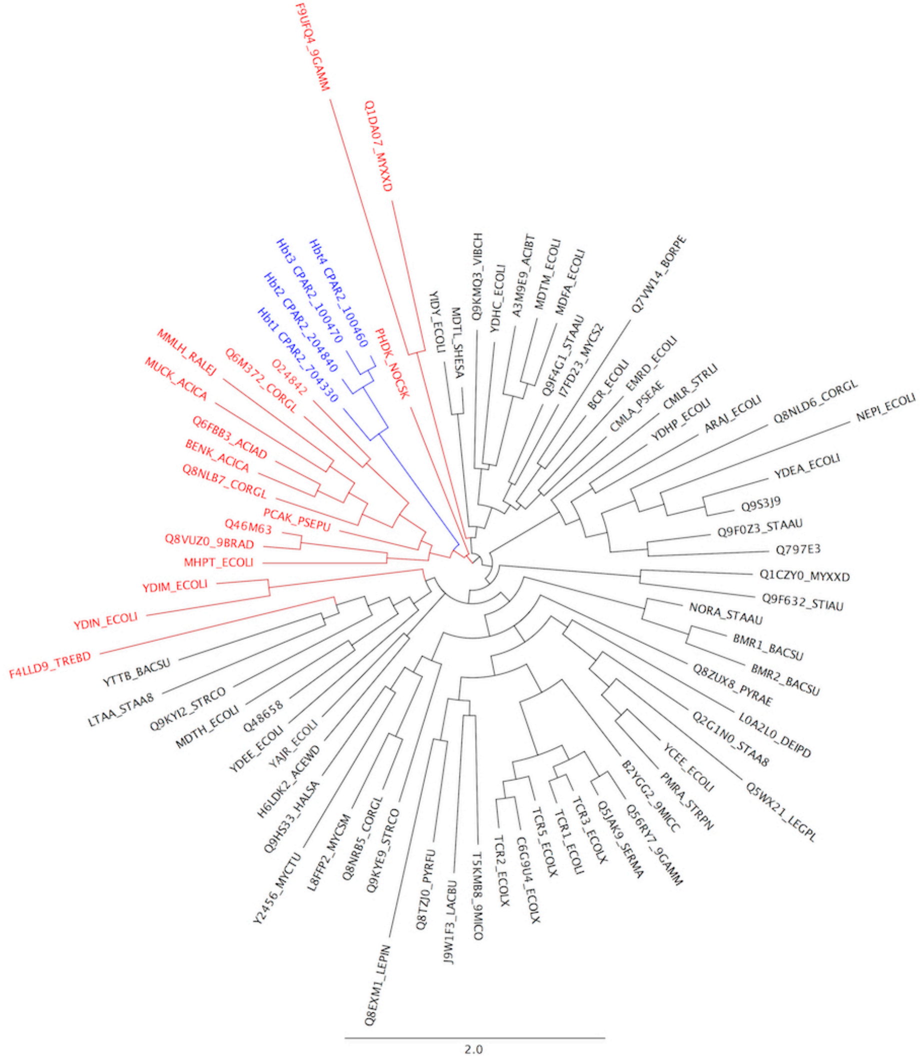

Supplementary Figure S3. Phylogenetic analysis of the AAHS (17 sequences, shown in red) and DHA1 (60 sequences, in black) transporters and the Hbt permeases from *C. parapsilosis* (in blue). The sequences of bacterial transporters were extracted from the TCDB database (http://www.tcdb.org/; as of July 1, 2017) and aligned using the MAFFT algorithm. Conserved regions of TMHs 2-3 and 8-9 regions were extracted from the alignment, concatenated and the columns containing gaps were omitted from the analysis. The tree was calculated using the PHYML algorithm and the LG substitution model. *C. parapsilosis* Hbt proteins represent a sister branch of typical AAHS permeases (*i.e.* BenK, GenK, MhpT, PcaK). Note that several proteins currently classified into AAHS family (*i.e.* F4LLD9_TREBD, F9UFQ4_9GAMM, Q1DA07_MYXXD, YDIM_ECOLI and YDIN_ECOLI) localized outside the main cluster formed by typical AAHS permeases are annotated as uncharacterized or putative and YDIM_ECOLI was shown to be involved in the efflux of isoprenol[7] indicating that the affiliation of these proteins to the AAHS family is uncertain.

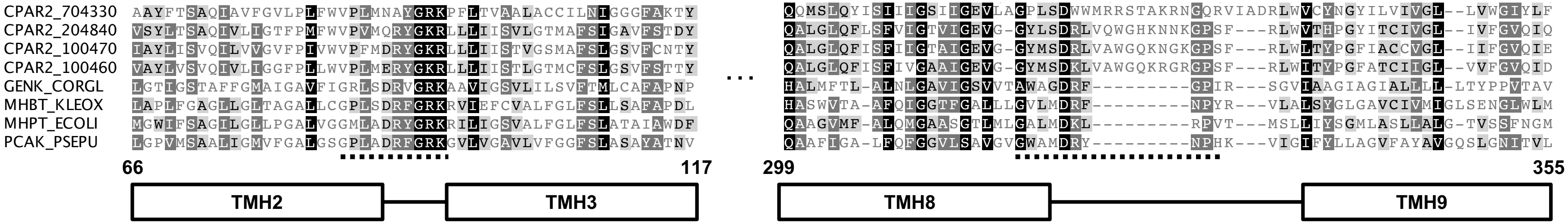

Supplementary Figure S4. Amino acid sequence alignments of the protein regions corresponding to the transmembrane helices TMH 2-3 and TMH 8-9 of *C. parapsilosis* Hbt transporters (Hbt1 = CPAR2_704330; Hbt2 = CPAR2_204840; Hbt3 = CPAR2_100470; Hbt4 = CPAR2_100460) and bacterial AAHS permeases GenK, MhbT, MhpT and PcaK. Sequences similar to the consensus motif 'GXXXD(R/K)XGR(R/K)' present between the helices in the bacterial AAHS proteins are underlined. The sequence numbering refers to PcaK from *Pseudomonas putida*.

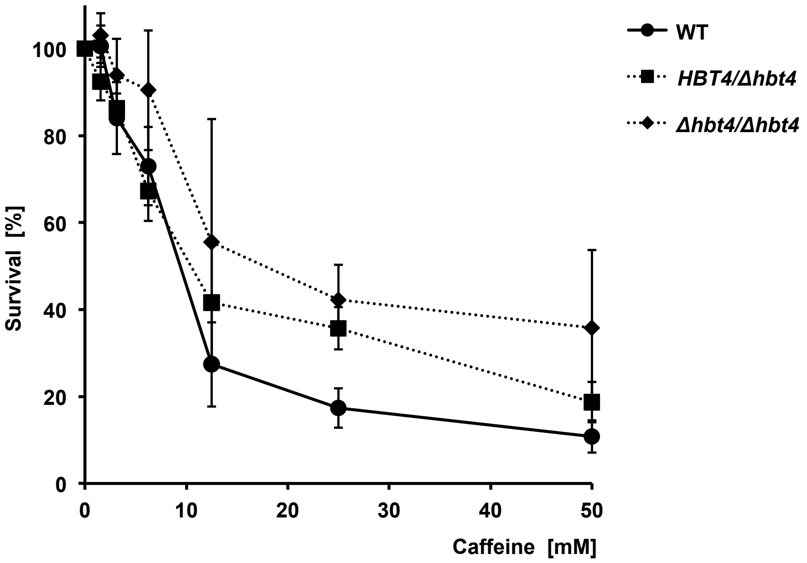

Supplementary Figure S5. The *C. parapsilosis* cells depleted for Hbt4 protein exhibit increased resistance to caffeine. The wild type, *HBT4/*Δ*hbt4* and Δ*hbt4/*Δ*hbt4* cells were grown in YPD medium containing increased concentrations of caffeine (0 – 50 mM) at 30 ^{o}C.

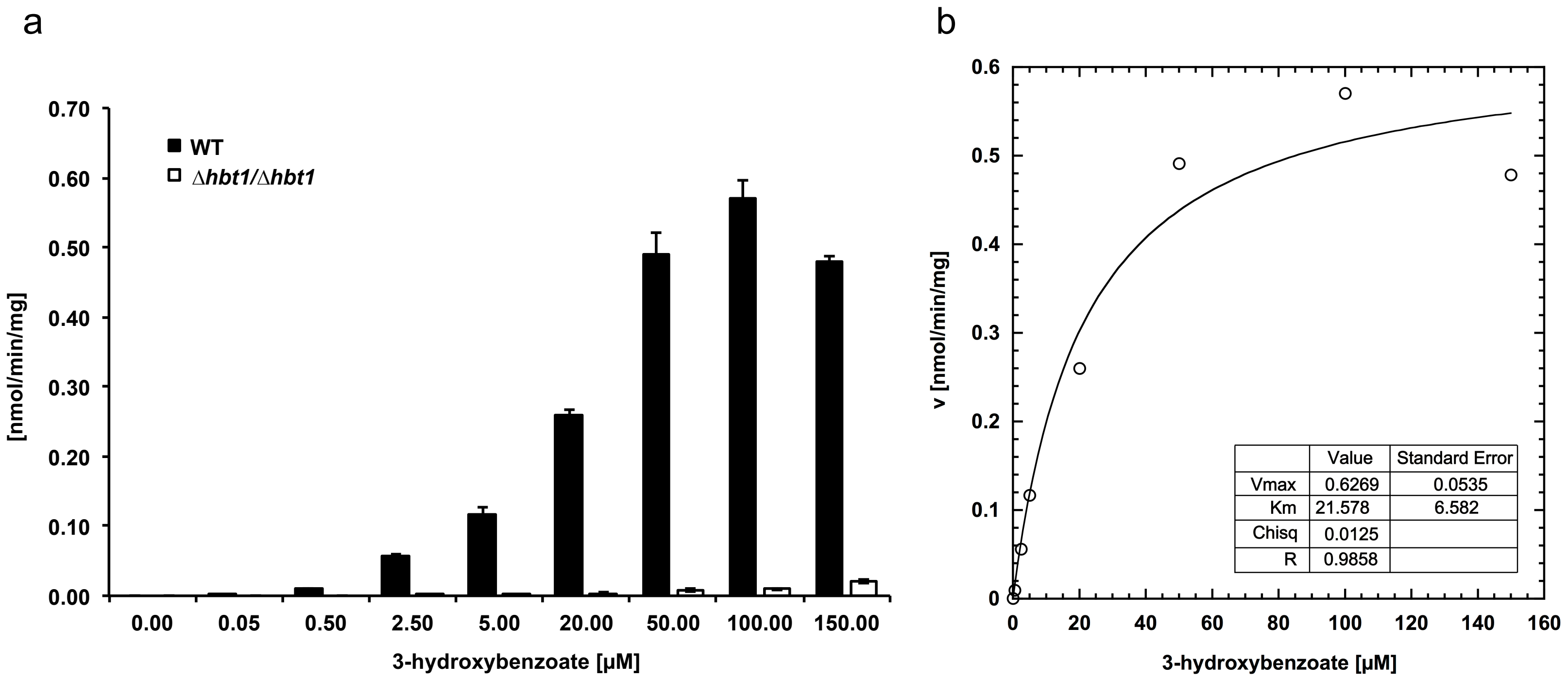

Supplementary Figure S6. The kinetics of the 3-hydroxybenzoate uptake by *C. parapsilosis* cells grown in S3OH medium. The uptake of $[^{14}C]$3-hydroxybenzoate was measured in triplicates at 1 min with eight substrate concentrations ranging from 0.05 to 150 µM. (a) Comparison of the uptake in the wild type and *Δhbt1/Δhbt1* cells. (b) Data obtained from mean values of the wild type cells were fitted with the Michaelis-Menten equation using a nonlinear fitting routine of KaleidaGraph software (Synergy Software, Reading, PA).

Supplementary References

1. Ditty, J. L. & Harwood, C. S. Charged amino acids conserved in the aromatic acid/H^+ symporter family of permeases are required for 4-hydroxybenzoate transport by PcaK from *Pseudomonas putida*. *J. Bacteriol.* **184**, 1444-1448 (2002).
2. Ditty, J. L. & Harwood, C. S. Conserved cytoplasmic loops are important for both the transport and chemotaxis functions of PcaK, a protein from *Pseudomonas putida* with 12 membrane-spanning regions. *J. Bacteriol.* **181**, 5068-5074 (1999).
3. Nichols, N. N. & Harwood, C. S. PcaK, a high-affinity permease for the aromatic compounds 4-hydroxybenzoate and protocatechuate from *Pseudomonas putida*. *J. Bacteriol.* **179**, 5056-5061 (1997).
4. Xu, Y. *et al.* MhbT is a specific transporter for 3-hydroxybenzoate uptake by Gram-negative bacteria. *Appl. Environ. Microbiol*. **78**, 6113-6120 (2012).
5. Xu, Y., Chen, B., Chao, H. & Zhou, N. Y. mhpT encodes an active transporter involved in 3-(3-hydroxyphenyl)propionate catabolism by *Escherichia coli* K-12. *Appl. Environ. Microbiol.* **79**, 6362-6368 (2013).
6. Xu, Y., Wang, S. H., Chao, H. J., Liu, S. J. & Zhou, N. Y. Biochemical and molecular characterization of the gentisate transporter GenK in *Corynebacterium glutamicum*. *PLoS One* **7**, e38701(2012).
7. Wang, C., Yang, L., Shah, A. A., Choi, E. S. & Kim, S. W. Dynamic interplay of multidrug transporters with TolC for isoprenol tolerance in *Escherichia coli*. *Sci. Rep*. **5,** 16505 (2015).

Supplementary File S1.

(Candida_SGZ55332.1:0.00662881,Candida_SGZ55445.1:0.00809682,
((Scheffersomyces_stipitis_CBS_6054_XP_001383098.2:0.11329825,Scheff
ersomyces_stipitis_CBS_6054_XP_001383641.2:0.32382461)1.000000:0.576
81185,
(((Debaryomyces_fabryi_XP_015464592.1:0.25803027,Debaryomyces_fabryi
_XP_015464494.1:0.21574422)1.000000:0.47635157,
((Debaryomyces_fabryi_XP_015465563.1:0.38956842,Debaryomyces_hanseni
i_CBS767_XP_002770238.1:0.38108718)1.000000:0.39652930,
((Kuraishia_capsulata_CBS_1993_CDK26262.1:0.76825105,Brettanomyces_b
ruxellensis_AWRI1499_EIF48744.1:1.42313935)0.999000:0.71433758,
((Sugiyamaella_lignohabitans_XP_018737106.1:0.00000001,Sugiyamaella_
lignohabitans_XP_018737106.1:0.00000001)0.000000:1.11578809,
((Nadsonia_fulvescens_var._elongata_DSM_6958_ODQ66882.1:0.03253031,N
adsonia_fulvescens_var._elongata_DSM_6958_ODQ66882.1:0.03253031)0.00
0000:0.81367910,
(((Wickerhamomyces_anomalus_NRRL_Y-366-8_XP_019037533.1:0.00000001,W
ickerhamomyces_anomalus_NRRL_Y-366-8_XP_019037533.1:0.00000001)0.000
000:1.40214375,
((Spathaspora_passalidarum_NRRL_Y-27907_XP_007375379.1:0.37377304,
(Lodderomyces_elongisporus_NRRL_YB-4239_XP_001525084.1:0.80366346,
((Candida_parapsilosis_CCE42841.1:0.08986078,Candida_orthopsilosis_X
P_003869470.1:0.04777455)1.000000:0.45516301,
((Candida_orthopsilosis_XP_003870416.1:0.02979555,Candida_parapsilos
is_CCE40008.1:0.28827575)1.000000:0.28605592,
(Candida_orthopsilosis_XP_003870415.1:0.04515605,Candida_parapsilosi
s_CCE40007.1:0.10376832)0.997000:0.18293926)0.994000:0.22513509)0.98
3000:0.28127741)0.937000:0.18060554)1.000000:1.01238860,
((Meyerozyma_guilliermondii_ATCC_6260_EDK41474.2:0.01520004,Meyerozy
ma_guilliermondii_ATCC_6260_XP_001482552.1:0.06207105)1.000000:0.578
36342,
((Debaryomyces_fabryi_XP_015466425.1:0.00000001,Debaryomyces_fabryi_
XP_015466425.1:0.00000001)0.000000:0.12073325,
(Debaryomyces_hansenii_CBS767_XP_002770429.1:0.00670059,Debaryomyces
_hansenii_CBS767_XP_002770429.1:0.00670059)0.000000:0.09736715)0.000
000:0.53111612)0.000000:0.60713726)0.000000:0.31192162)1.000000:0.44
445251,
((((Pseudogymnoascus_sp._VKM_F-4514__FW-929__KFY42708.1:0.00592900,P
seudogymnoascus_sp._VKM_F-3808_KFX99388.1:0.08463445)0.779000:0.0379
1566,(Pseudogymnoascus_sp._05NY08_OBT70736.1:0.05808693,
(Pseudogymnoascus_sp._VKM_F-4246_KFY17534.1:0.02196544,
(Pseudogymnoascus_sp._03VT05_OBT89218.1:0.01118736,
(Pseudogymnoascus_sp._VKM_F-4519__FW-2642__KFZ17943.1:0.00559217,
(Pseudogymnoascus_sp._VKM_F-103_KFY80242.1:0.00284297,Pseudogymnoasc
us_verrucosus_XP_018126051.1:0.10841898)0.909000:0.00829214)0.756000
:
0.00267830)0.886000:0.01042483)0.952000:0.02391681)0.861000:0.040676
31)1.000000:1.02632335,
((Aspergillus_tubingensis_CBS_134.48_OJI87284.1:0.10096054,Aspergill
us_niger_GAQ33186.1:0.04602341)1.000000:1.06387688,
(((((Penicillium_subrubescens_OKO97877.1:0.26887031,
(Penicillium_camemberti_CRL26834.1:0.29432003,Penicillium_arizonense
_OGE47732.1:0.20339070)0.986000:0.15379117)1.000000:0.36206968,

(Aspergillus_ruber_CBS_135680_EYE98774.1:0.05626618,Aspergillus_glau
cus_CBS_516.65_OJJ87943.1:0.02431030)1.000000:0.57152668)0.261000:0.
07151647,Aspergillus_wentii_DTO_134E9_OJJ34711.1:0.47643250)0.808000
:0.09123326,
((Aspergillus_nomius_NRRL_13137_XP_015402538.1:0.20813085,
(Aspergillus_parasiticus_SU-1_KJK65859.1:0.03526085,
(Aspergillus_flavus_AF70_KOC08732.1:0.00000070,Aspergillus_flavus_NR
RL3357_XP_002375448.1:0.06844618)0.943000:0.04664571)0.937000:0.0939
7684)1.000000:0.42764599,
(Aspergillus_clavatus_NRRL_1_XP_001268553.1:0.66931215,
(Aspergillus_calidoustus_CEN60399.1:0.60103545,
(Aspergillus_terreus_NIH2624_XP_001218672.1:0.34957262,
(Aspergillus_aculeatus_ATCC_16872_XP_020060677.1:0.34353389,
(Aspergillus_carbonarius_ITEM_5010_OOF99267.1:0.20581702,
((Aspergillus_luchuensis_CBS_106.47_OJZ81071.1:0.01460386,Aspergillu
s_luchuensis_GAT22017.1:0.02164044)0.976000:0.04046282,
((Aspergillus_niger_ATCC_1015_EHA19298.1:0.00000022,
(Aspergillus_niger_CBS_513.88_XP_001394042.2:0.00000001,Aspergillus_
niger_CAK48271.1:0.00000011)0.822000:0.00576136)0.998000:0.07331770,
Aspergillus_brasiliensis_CBS_101740_OJJ77467.1:0.11185179)0.030000:0
.
01418037)1.000000:0.15400344)0.016000:0.05898070)0.996000:0.18381195
)0.484000:0.06184004)0.469000:0.07065390)0.037000:0.04002061)0.72700
0:0.07562847)0.993000:0.22264038,
(Byssochlamys_spectabilis_No._5_GAD99525.1:0.46329509,
(Penicillium_griseofulvum_KXG51500.1:0.22538036,
(Penicillium_chrysogenum_KZN89041.1:0.00000001,Penicillium_rubens_Wi
sconsin_54-1255_XP_002568436.1:0.00000044)0.962000:0.09869606)1.0000
00:0.35932518)1.000000:0.34299659)0.563000:0.12795614)0.994000:0.345
96588)0.998000:0.25464085,
((Byssochlamys_spectabilis_No._5_GAD97452.1:0.00000009,Byssochlamys_
spectabilis_No._5_GAD97452.1:0.00000009)0.000000:0.57018442,
((Glarea_lozoyensis_ATCC_20868_XP_008086202.1:0.59518931,
((Phialocephala_scopiformis_XP_018063897.1:0.14382516,Phialocephala_
subalpina_CZR55677.1:0.20240405)0.996000:0.22410799,
(Pseudogymnoascus_sp._WSF_3629_OBT38975.1:0.37619504,
(Oidiodendron_maius_Zn_KIN02246.1:0.04566644,Oidiodendron_maius_Zn_K
IM95798.1:0.14328009)0.995000:0.17947537)0.997000:0.20285481)0.85900
0:0.13979240)1.000000:0.28010037,
((Verruconis_gallopava_XP_016211393.1:0.77871593,
(Lepidopterella_palustris_CBS_459.81_OCK78496.1:0.36959699,
((Diplodia_seriata_KKY22838.1:0.00000010,Diplodia_seriata_KKY22838.1
:0.00000010)0.000000:0.23938059,
(Neofusicoccum_parvum_UCRNP2_XP_007581166.1:0.17091239,
(Macrophomina_phaseolina_MS6_EKG15541.1:0.00000010,Macrophomina_phas
eolina_MS6_EKG15541.1:0.00000010)0.000000:0.11025700)0.698000:0.0931
4878)0.289000:0.14242941)0.000000:0.08640808)0.964000:0.13857598,
((Baudoinia_panamericana_UAMH_10762_XP_007678008.1:0.37369064,
(Dothistroma_septosporum_NZE10_EME48374.1:0.00233384,Dothistroma_sep
tosporum_NZE10_EME48374.1:0.00233384)0.000000:0.43887194)0.809000:0.
22217043,
((Aureobasidium_melanogenum_CBS_110374_KEQ67594.1:0.07049454,
(Aureobasidium_subglaciale_EXF-2481_XP_013344290.1:0.05384835,Aureob
asidium_namibiae_CBS_147.97_XP_013431575.1:0.08003493)0.944000:0.071

48642)1.000000:0.41895503,
(((Rasamsonia_emersonii_CBS_393.64_XP_013330495.1:0.33448259,
((Talaromyces_islandicus_CRG92661.1:0.31376969,
(Talaromyces_atroroseus_XP_020122458.1:0.36450995,
(Talaromyces_stipitatus_ATCC_10500_XP_002486650.1:0.10694771,
(Talaromyces_verruculosus_KUL89161.1:0.13202390,
(Talaromyces_marneffei_PM1_KFX42089.1:0.11287550,Talaromyces_marneffei_ATCC_18224_XP_002152902.1:0.01966138)0.949000:0.06695756)0.000000:
0.03292869)0.995000:0.16203523)0.698000:0.11627512)0.975000:0.13653714,Talaromyces_cellulolyticus_GAM34922.1:0.72119401)0.044000:0.02822927)0.997000:0.21214544,
(((Penicillium_arizonense_OGE47545.1:0.12208758,
(((Penicillium_nordicum_KOS38834.1:0.00000001,Penicillium_nordicum_KOS38834.1:0.00000001)0.000000:0.03750678,
((Penicillium_camemberti_CRL18343.1:0.00000001,Penicillium_camemberti_CRL18343.1:0.00000001)0.000000:0.01989447,
(Penicillium_freii_KUM62748.1:0.00555635,Penicillium_freii_KUM62748.1:0.00555635)0.000000:0.03157166)0.000000:0.01089634)0.961000:0.03443071,
(((Penicillium_chrysogenum_XP_002566086.1:0.00000012,Penicillium_rubens_Wisconsin_54-1255_XP_002566086.1:0.00577153)0.999000:0.05690012,
(Penicillium_roqueforti_FM164_CDM27469.1:0.00568038,Penicillium_roqueforti_FM164_CDM27469.1:0.00568038)0.925000:0.07613704)0.708000:0.00359944,
(((Penicillium_expansum_XP_016602999.1:0.00000001,Penicillium_expansum_XP_016602999.1:0.00000001)0.000000:0.00278301,
(Penicillium_expansum_KGO54491.1:0.00000001,Penicillium_expansum_KGO54491.1:0.00000001)0.858000:0.00000014)0.739000:0.01469551,
(Penicillium_italicum_KGO77709.1:0.00497824,Penicillium_italicum_KGO77709.1:0.00497824)0.000000:0.07095418)0.913000:0.01766388)0.484000:
0.01769954)0.997000:0.12180930)1.000000:0.21634160,
(((Aspergillus_carbonarius_ITEM_5010_OOF95902.1:0.00000001,Aspergillus_carbonarius_ITEM_5010_OOF95902.1:0.00000001)0.000000:0.06242840,
((Aspergillus_brasiliensis_CBS_101740_OJJ74904.1:0.00000001,Aspergillus_brasiliensis_CBS_101740_OJJ74904.1:0.00000001)0.000000:0.04224485,
((Aspergillus_niger_ATCC_1015_EHA26493.1:0.00000001,Aspergillus_niger_ATCC_1015_EHA26493.1:0.00000001)0.000000:0.02269691,
(Aspergillus_tubingensis_CBS_134.48_OJI86908.1:0.00290109,
((Aspergillus_luchuensis_CBS_106.47_GAQ33792.1:0.00000001,Aspergillus_niger_GAQ33792.1:0.00000001)0.000000:0.00000012,
(Aspergillus_kawachii_IFO_4308_GAA83518.1:0.00000001,Aspergillus_kawachii_IFO_4308_GAA83518.1:0.00000001)0.715000:0.00289607)0.000000:0.00000013)0.272000:0.01600844)0.997000:0.03636602)0.775000:0.07832891)0.998000:0.24863098,((Aspergillus_cristatus_ODM21943.1:0.02199614,
(Aspergillus_glaucus_CBS_516.65_OJJ83565.1:0.02679990,Aspergillus_ruber_CBS_135680_EYE95268.1:0.05160456)0.742000:0.02649009)1.000000:0.21440250,(Aspergillus_bombycis_OGM45711.1:0.02158607,
(Aspergillus_parasiticus_SU-1_KJK63096.1:0.01688598,
(Aspergillus_oryzae_100-8_EIT80122.1:0.00000001,Aspergillus_oryzae_BAE55871.1:0.00307991)0.935000:0.01130708)0.910000:0.02174241)0.999000:0.14508006)0.984000:0.09448927)0.991000:0.19279670)0.999000:0.26316171,((Aspergillus_wentii_DTO_134E9_OJJ31051.1:0.30129904,

((Aspergillus_udagawae_GAO86836.1:0.04196532,
(Aspergillus_fumigatus_Af293_XP_755225.1:0.02712604,Aspergillus_fisc
heri_NRRL_181_XP_001260394.1:0.01586231)0.883000:0.02157668)1.000000
:0.24147525,
(((Aspergillus_aculeatus_ATCC_16872_XP_020059866.1:0.00290153,Asperg
illus_aculeatus_ATCC_16872_XP_020059866.1:0.00290153)0.000000:0.1528
1850,
((Aspergillus_carbonarius_ITEM_5010_OOF92471.1:0.00000016,Aspergillu
s_carbonarius_ITEM_5010_OOF92471.1:0.00000016)0.000000:0.06927601,
((Aspergillus_brasiliensis_CBS_101740_OJJ67343.1:0.00000001,Aspergil
lus_brasiliensis_CBS_101740_OJJ67343.1:0.00000001)1.000000:0.0837106
8,((Aspergillus_niger_CAK97152.1:0.00000001,
(Aspergillus_niger_CAK97152.1:0.00000001,
(Aspergillus_niger_ATCC_1015_EHA25236.1:0.00000001,Aspergillus_niger
_ATCC_1015_EHA25236.1:0.00000001)0.000000:0.00269867)0.916000:0.0000
0009)0.000000:0.01494677,
(Aspergillus_tubingensis_CBS_134.48_OJI80582.1:0.03186979,
(Aspergillus_tubingensis_CBS_134.48_OJI80582.1:0.00000014,
(Aspergillus_luchuensis_CBS_106.47_GAA86474.1:0.00000001,Aspergillus
_kawachii_IFO_4308_GAA86474.1:0.00000001)0.776000:0.00545810)0.90700
0:0.00085957)0.000000:0.01997928)0.000000:0.02782545)0.972000:0.0333
2830)0.833000:0.02827927)0.959000:0.14339140,
((Aspergillus_flavus_AF70_KOC08616.1:0.00269501,Aspergillus_oryzae_R
IB40_XP_003190829.1:0.00000012)1.000000:0.27502469,
(Aspergillus_calidoustus_CEL01070.1:0.34321918,
(Aspergillus_sydowii_CBS_593.65_OJJ58753.1:0.04589992,Aspergillus_ve
rsicolor_CBS_583.65_OJJ01361.1:0.04540634)0.979000:0.08156533)0.9970
00:0.16199479)0.882000:0.08237102)0.000000:0.04260033)0.000000:0.050
72608)1.000000:0.19623590,
(Talaromyces_atroroseus_XP_020118403.1:0.59650968,
((Aspergillus_calidoustus_CEL05877.1:0.00000001,Aspergillus_calidous
tus_CEL05877.1:0.00000001)0.000000:0.41126019,
((Penicillium_arizonense_OGE50785.1:0.25799962,
(Aspergillus_glaucus_CBS_516.65_OJJ88214.1:0.52339644,
((Aspergillus_oryzae_RIB40_XP_001818692.2:0.00000001,Aspergillus_ory
zae_OOO09432.1:0.00289090)0.998000:0.06677351,
(Aspergillus_nomius_NRRL_13137_XP_015411287.1:0.04913986,Aspergillus
_bombycis_OGM48237.1:0.02436775)0.959000:0.04051669)0.995000:0.15414
146)0.524000:0.05135811)0.888000:0.04799686,
(Aspergillus_aculeatus_ATCC_16872_XP_020054849.1:0.30651088,
((Aspergillus_niger_ATCC_1015_CAK44163.1:0.00000001,Aspergillus_nige
r_CBS_513.88_XP_001389854.2:0.00000012)0.902000:0.02784258,
(Aspergillus_luchuensis_CBS_106.47_OJZ80660.1:0.01216155,
(Aspergillus_niger_GAQ46897.1:0.00000001,Aspergillus_tubingensis_CBS
_134.48_OJI87516.1:0.00000012)0.832000:0.00687175)0.787000:0.0133722
6)1.000000:0.24188763)0.881000:0.08501290)0.977000:0.03388848)0.0000
00:0.09544479)0.999000:0.24249131)0.000000:0.07353636)0.857000:0.067
71440)0.858000:0.09296249,
((Pseudogymnoascus_sp._03VT05_OBT90698.1:0.52303072,
(Phialocephala_scopiformis_XP_018062177.1:0.32157959,
(Botrytis_cinerea_BcDW1_XP_001553898.1:0.22195330,Sclerotinia_sclero
tiorum_1980_UF-70_XP_001590814.1:0.19837670)1.000000:0.26714869)0.97
1000:0.16450774)0.990000:0.13441199,
(((Fonsecaea_multimorphosa_XP_016628463.1:0.42557571,Exophiala_xenob

iotica_XP_013318565.1:0.33155133)0.906000:0.12333681,
(Ophiostoma_piceae_UAMH_11346_EPE06512.1:0.75250647,
((Macrophomina_phaseolina_MS6_EKG18724.1:0.47467371,
(Verruconis_gallopava_XP_016214252.1:0.63933208,Coniochaeta_ligniari
a_NRRL_30616_OIW34601.1:0.65506985)0.766000:0.09490362)0.957000:0.11
694635,(Pestalotiopsis_fici_W106-1_XP_007829615.1:0.92730669,
((Aspergillus_niger_ATCC_1015_EHA26721.1:0.00072354,Aspergillus_nige
r_CAK37115.1:0.08631704)1.000000:0.65028850,
(Penicillium_arizonense_OGE49036.1:0.12817103,
(Penicillium_roqueforti_FM164_CDM29962.1:0.15302508,
(Penicillium_griseofulvum_KXG51704.1:0.12461653,
(Penicillium_expansum_XP_016595214.1:0.05302479,
((Penicillium_rubens_Wisconsin_54-1255_XP_002560492.1:0.16746395,Pen
icillium_camemberti_CRL20018.1:0.01725187)0.341000:0.00716903,Penici
llium_freii_KUM59931.1:0.04135904)0.894000:0.01396320)0.794000:0.010
09261)0.091000:0.02116681)0.997000:0.13773378)1.000000:0.33334905)0.
336000:0.07478622)0.212000:0.05136222)0.746000:0.03006694)0.983000:0
.15990761)0.996000:0.18947405,
(Glonium_stellatum_OCL03718.1:0.33587403,
((Paraphaeosphaeria_sporulosa_XP_018029543.1:0.02993469,Paraphaeosph
aeria_sporulosa_XP_018029543.1:0.02993469)1.000000:0.43233590,
(((Stagonospora_sp._SRC1lsM3a_OAK97258.1:0.18571056,
(Bipolaris_victoriae_FI3_XP_007708445.1:0.27708173,
(Pyrenophora_teres_f._teres_0-1_XP_003301074.1:0.03520498,Pyrenophor
a_tritici-repentis_Pt-1C-
BFP_XP_001939687.1:0.03936193)0.999000:0.11001445)0.992000:0.1092705
1)0.530000:0.06572549,Ascochyta_rabiei_KZM21248.1:0.23865807)0.57300
0:0.06422296,Pyrenochaeta_sp._DS3sAY3a_OAL48256.1:0.17016167)0.99400
0:0.14894789)0.999000:0.26410477)0.797000:0.03873875)0.892000:0.0673
2382)0.903000:0.05231107)0.958000:0.09331235)1.000000:0.27376782)0.0
00000:0.07777138)0.747000:0.21242315)0.000000:0.17559468)1.000000:0.
56485637)0.000000:0.30443045)0.623000:0.16235695)0.000000:0.13582311
)1.000000:0.30649541)0.988000:0.26427811)0.000000:0.12393975)0.84600
0:0.16233105,
(Lodderomyces_elongisporus_NRRL_YB-4239_XP_001524534.1:0.93674644,
(Candida_maltosa_Xu316_EMG48866.1:0.39475462,
(Candida_parapsilosis_CCE45419.1:0.19885789,Spathaspora_passalidarum
_NRRL_Y-27907_XP_007372975.1:0.27019071)0.990000:0.16937010)0.792000
:
0.06243238)0.972000:0.13544373)0.970000:0.18369544)1.000000:1.458628
60);

Supplementary File S2.

(Ogataea_polymorpha_XP_018212756.1:0.06130369,Ogataea_parapolymorpha
_DL-1_XP_013933058.1:0.08447729,
(Babjeviella_inositovora_NRRL_Y-12698_XP_018988586.1:0.80454766,
(((Candida_SGZ53569.1:0.00000522,Candida_SGZ58333.1:0.00477584)1.000
000:1.09158044,
((Debaryomyces_hansenii_CBS767_XP_461135.2:0.12174202,Debaryomyces_f
abryi_XP_015469007.1:0.16367492)1.000000:0.48916689,
(Scheffersomyces_stipitis_CBS_6054_XP_001386445.2:0.54779668,
(Spathaspora_passalidarum_NRRL_Y-27907_XP_007376166.1:0.48394162,
(Lodderomyces_elongisporus_NRRL_YB-4239_XP_001527239.1:0.48572296,
(Candida_parapsilosis_CCE40241.1:0.08518914,Candida_orthopsilosis_Co
_90-125_XP_003867550.1:0.13831992)0.999000:0.50110335)1.000000:0.642
38602)0.991000:0.34727380)0.888000:0.20330539)0.474000:0.16319421)0.
999000:0.60667051,
((Wickerhamomyces_anomalus_NRRL_Y-366-8_XP_019040271.1:0.01261547,Wi
ckerhamomyces_anomalus_NRRL_Y-366-8_XP_019040178.1:0.00775716)1.0000
00:0.96287401,(Sugiyamaella_lignohabitans_XP_018735344.1:1.49052439,
(((Tuber_melanosporum_Mel28_XP_002836887.1:1.19466228,Pyronema_ompha
lodes_CBS_100304_CCX31605.1:1.09442596)0.632000:0.16475964,
(Pyronema_omphalodes_CBS_100304_CCX06863.1:1.51177772,
(Pseudogymnoascus_sp._VKM_F-3775_KFY23046.1:1.52779844,
((((Glonium_stellatum_OCL07385.1:0.51425334,
(Macrophomina_phaseolina_MS6_EKG10154.1:0.41294184,Diplodia_corticol
a_XP_020133541.1:0.24260797)1.000000:0.52323502)0.966000:0.23568043,
(Coniosporium_apollinis_CBS_100218_XP_007784127.1:0.72320697,
(Exophiala_xenobiotica_XP_013309833.1:1.05023318,Sphaerulina_musiva_
SO2202_XP_016758231.1:1.09183544)0.333000:0.15188561)0.214000:0.1063
7521)0.950000:0.21247863,
(Verruconis_gallopava_XP_016210604.1:0.82081506,
(Aspergillus_aculeatus_ATCC_16872_XP_020051682.1:0.39656048,
(Aspergillus_wentii_DTO_134E9_OJJ33792.1:0.31959138,
(Aspergillus_flavus_AF70_KOC14394.1:0.00000033,
(Aspergillus_oryzae_RIB40_XP_003190342.1:0.00000016,Aspergillus_oryz
ae_OOO06416.1:0.00000015)0.707000:0.00425225)1.000000:0.31852071)0.7
22000:0.11826273)0.995000:0.26153781)0.984000:0.27838564)0.080000:0.
06386866,
((Microdochium_bolleyi_KXJ85635.1:0.77254749,Metarhizium_acridum_CQM
a_102_XP_007814564.1:0.52121467)0.995000:0.31977280,
(((Neonectria_ditissima_KPM38498.1:0.55400521,
(Pseudogymnoascus_sp._VKM_F-4515__FW-2607__KFY47532.1:0.21586903,
((Pseudogymnoascus_sp._03VT05_OBT87707.1:0.12233299,Pseudogymnoascus
_sp._05NY08_OBT80483.1:0.06148508)0.907000:0.08760821,
(Pseudogymnoascus_sp._VKM_F-4519__FW-2642__KFZ06304.1:0.01394601,
(Pseudogymnoascus_verrucosus_XP_018126599.1:0.02276276,Pseudogymnoas
cus_sp._VKM_F-103_KFY77496.1:0.00570541)0.939000:0.03506345)0.415000
:
0.05109892)0.919000:0.11247752)1.000000:0.58629718)0.948000:0.163565
89,Scedosporium_apiospermum_XP_016645580.1:0.91782945)0.694000:0.059
57642,(Paraphaeosphaeria_sporulosa_XP_018042804.1:0.85637350,
(((Valsa_mali_KUI67902.1:0.76719547,
(Colletotrichum_chlorophyti_OLN92755.1:0.23070733,
(Colletotrichum_simmondsii_KXH25333.1:0.00000001,Colletotrichum_fior

iniae_PJ7_XP_007600666.1:0.00399818)0.999000:0.23834251)1.000000:0.2
9353360)0.762000:0.07276423,Pestalotiopsis_fici_W106-1_XP_007830685.
1:0.46488724)0.831000:0.06640092,
((Nectria_haematococca_mpVI_77-13-4_XP_003042150.1:0.82037965,
(Phaeoacremonium_minimum_UCRPA7_XP_007913422.1:0.56423358,Eutypa_lat
a_UCREL1_XP_007796017.1:0.47081332)0.749000:0.09400343)0.837000:0.13
790198,(Phialophora_attae_XP_017999181.1:0.60127640,
(Verticillium_alfalfae_VaMs.
102_XP_003007891.1:0.00776543,Verticillium_longisporum_CRK31428.1:0.
06040264)1.000000:0.57703489)0.939000:0.15132247)0.238000:0.05743365
)0.348000:0.08714902)0.643000:0.07510523)0.925000:0.16988557)0.97100
0:0.20923960)0.975000:0.54165528)1.000000:1.77306744)0.706000:0.3901
5808)0.497000:0.12545204,
((Boreostereum_vibrans_ANB32222.1:0.03519119,Stereum_hirsutum_FP-916
66_SS1_XP_007305833.1:0.09309312)1.000000:0.80553659,
((Glonium_stellatum_OCL12374.1:0.62487008,
((Arthroderma_otae_CBS_113480_XP_002851307.1:0.23013854,
(Nannizzia_gypsea_CBS_118893_XP_003177893.1:0.13147205,
((Trichophyton_violaceum_OAL70860.1:0.00000001,
(Trichophyton_rubrum_CBS_118892_XP_003235631.1:0.00000001,
(Trichophyton_rubrum_MR1459_EZF94478.1:0.22231564,Trichophyton_rubru
m_CBS_202.88_EZG16018.1:0.07043509)0.000000:0.00003578)0.775000:0.03
734243)0.972000:0.04909245,
((Trichophyton_benhamiae_CBS_112371_XP_003011078.1:0.01101901,Tricho
phyton_verrucosum_HKI_0517_XP_003020161.1:0.00547647)0.884000:0.0113
9567,(Trichophyton_interdigitale_MR816_KDB24625.1:0.00000017,
(Trichophyton_equinum_CBS_127.97_EGE08271.1:0.00000001,Trichophyton_
tonsurans_CBS_112818_EGD98775.1:0.00567822)0.809000:0.00546185)0.979
000:0.03851923)0.372000:0.01438291)0.998000:0.15591603)0.877000:0.12
985164)1.000000:0.97242510,
(Uncinocarpus_reesii_1704_XP_002585004.1:0.18943657,
(Coccidioides_posadasii_C735_delta_SOWgp_XP_003070346.1:0.00557201,
(Coccidioides_posadasii_str._Silveira_EFW23391.1:0.00000001,
((Coccidioides_immitis_RS_XP_001243320.1:0.00000001,Coccidioides_imm
itis_RMSCC_2394_KMP04398.1:0.06277955)0.000000:0.00000001,Coccidioid
es_immitis_H538.4_KMU91067.1:0.03591786)0.945000:0.01686836)0.000000
:
0.00000001)0.965000:0.12763275)0.988000:0.28335507)0.984000:0.281459
45)0.697000:0.14549222,
(((Exophiala_xenobiotica_XP_013315334.1:0.50443783,Rhinocladiella_ma
ckenziei_CBS_650.93_XP_013277369.1:0.64966764)1.000000:1.22003213,
(Oidiodendron_maius_Zn_KIM92686.1:0.22431044,
(Phialocephala_subalpina_CZR67538.1:0.31646691,
(Oidiodendron_maius_Zn_KIN03572.1:0.51757486,Oidiodendron_maius_Zn_K
IN07030.1:0.31408763)1.000000:0.41617748)0.451000:0.15508438)1.00000
0:1.20254422)0.848000:0.29116858,
(((Exophiala_sideris_KIV79256.1:0.31771859,
(Exophiala_xenobiotica_XP_013310826.1:0.26508680,
(Exophiala_spinifera_XP_016235709.1:0.25258051,Exophiala_oligosperma
_XP_016264715.1:0.22174852)0.998000:0.19291283)0.980000:0.14634830)0
.998000:0.21966627,
(Rhinocladiella_mackenziei_CBS_650.93_XP_013273572.1:0.27623119,
(Exophiala_dermatitidis_NIH/UT8656_XP_009154952.1:0.28891683,
(Capronia_coronata_CBS_617.96_XP_007724951.1:0.14451111,Capronia_epi

myces_CBS_606.96_XP_007729452.1:0.19051718)0.848000:0.06619707)0.983
000:0.13798054)0.776000:0.11626796)0.862000:0.05689581,
((Phialophora_americana_KIW72870.1:0.10870188,
((Cladophialophora_carrionii_OCT44098.1:0.13431067,Cladophialophora_
carrionii_CBS_160.54_XP_008722660.1:0.12939024)0.999000:0.17191467,C
ladophialophora_yegresii_CBS_114405_XP_007753201.1:0.17029901)0.8620
00:0.04388318)0.997000:0.18320830,
(((Cladophialophora_psammophila_CBS_110553_XP_007745161.1:0.05072249
,Cladophialophora_bantiana_CBS_173.52_XP_016615286.1:0.07273163)0.99
9000:0.16907800,(Fonsecaea_erecta_XP_018690955.1:0.16747801,
(Fonsecaea_multimorphosa_CBS_102226_XP_016635632.1:0.00000001,Fonsec
aea_multimorphosa_OAL28270.1:0.01948682)0.999000:0.15989735)0.804000
:0.04975150)0.769000:0.04377209,
(Cladophialophora_immunda_XP_016255425.1:0.15368220,
(Fonsecaea_nubica_OAL40169.1:0.01852173,
(Fonsecaea_pedrosoi_CBS_271.37_XP_013281919.1:0.00000016,Fonsecaea_m
onophora_OAG39568.1:0.00557145)0.826000:0.01546052)1.000000:0.191998
94)0.989000:0.12682899)0.943000:0.10472467)0.874000:0.06265552)0.997
000:0.44480095)0.855000:0.11604242)0.952000:0.22854223)0.975000:0.30
392870)0.998000:0.63321976)0.990000:0.47915144)0.628000:0.12971969)0
.838000:0.16027174)1.000000:2.12642037);

Supplementary File S3.

(Botryobasidium_botryosum_FD-172_SS1_KDQ19543.1:0.05111415,Botryobas
idium_botryosum_FD-172_SS1_KDQ18167.1:0.04674434,
((Dacryopinax_primogenitus_EJU02716.1:0.23120362,Calocera_cornea_HHB
12733_KZT51075.1:0.18288372)0.995000:0.44712713,
(((Cladophialophora_immunda_XP_016246673.1:1.23456618,Exophiala_olig
osperma_XP_016261888.1:0.78298776)0.995000:0.64673567,
(Endocarpon_pusillum_Z07020_XP_007801460.1:2.35192127,
((Pyronema_omphalodes_CBS_100304_CCX33496.1:0.63345930,
(((Lichtheimia_ramosa_CDS10179.1:0.02965906,Lichtheimia_corymbifera_
JMRC:0.04513489)1.000000:0.43255236,
(Parasitella_parasitica_CEP11899.1:0.08145671,
(Mucor_ambiguus_GAN07197.1:0.01754311,
(Mucor_circinelloides_f._lusitanicus_CBS_277.49_OAC98245.1:0.0207583
8,Mucor_circinelloides_f._circinelloides_1006PhL_EPB84375.1:0.038704
66)0.121000:0.01308190)0.363000:0.02778961)1.000000:0.31568957)0.999
000:0.29204773,
(Sugiyamaella_lignohabitans_XP_018737107.1:0.52823928,
(Candida_tropicalis_MYA-3404_XP_002549620.1:0.65999380,
((Kuraishia_capsulata_CBS_1993_CDK26261.1:0.69473861,
(Spathaspora_passalidarum_NRRL_Y-27907_XP_007372976.1:0.17729223,
(Candida_parapsilosis_CCE45418.1:0.07053402,Candida_orthopsilosis_Co
_90-125_XP_003865847.1:0.16397020)0.966000:0.09289268)0.942000:0.110
43242)0.688000:0.06478312,
((Scheffersomyces_stipitis_CBS_6054_XP_001382556.1:0.10006279,Scheff
ersomyces_stipitis_CBS_6054_XP_001383962.1:0.14706850)1.000000:0.342
39224,
(Debaryomyces_fabryi_XP_015464493.1:0.10372444,Debaryomyces_fabryi_X
P_015464593.1:0.18661196)1.000000:0.23056121)0.484000:0.04462567)0.7
91000:0.14765649)0.649000:0.07613164)1.000000:0.39317484)0.853000:0.
17622184)0.952000:0.18747348,
((Phialocephala_subalpina_CZR62875.1:0.30056161,Phialocephala_scopif
ormis_XP_018063617.1:0.25715714)1.000000:0.85275087,
(((Rasamsonia_emersonii_CBS_393.64_XP_013328274.1:0.44357188,
(Uncinocarpus_reesii_1704_XP_002541886.1:0.21090004,
((Coccidioides_immitis_RMSCC_2394_KMP09917.1:0.00000001,
(Coccidioides_immitis_H538.4_KMU89437.1:0.62881541,
(Coccidioides_posadasii_RMSCC_3488_KMM64156.1:0.04354977,Coccidioide
s_posadasii_C735_delta_SOWgp_XP_003070875.1:0.00000026)0.733000:0.00
713339)0.000000:0.00000336)0.000000:0.00000001,Coccidioides_immitis_
RS_XP_001248580.2:0.00000017)1.000000:0.26760296)1.000000:0.34667778
)0.988000:0.18924464,
((Coniosporium_apollinis_CBS_100218_XP_007780425.1:0.56082730,
(Nectria_haematococca_mpVI_77-13-4_XP_003044092.1:0.36371977,Nectria
_haematococca_mpVI_77-13-4_XP_003049298.1:0.42103567)0.963000:0.1656
1607)0.993000:0.23111566,
(Exophiala_aquamarina_CBS_119918_XP_013265651.1:0.72685204,Exophiala
_aquamarina_CBS_119918_XP_013258072.1:0.39617586)0.996000:0.33482223
)0.894000:0.10773044)0.795000:0.07081101,
(Acremonium_chrysogenum_ATCC_11550_KFH46194.1:0.74559942,
(Purpureocillium_lilacinum_OAQ77829.1:0.00000091,Purpureocillium_lil
acinum_XP_018176021.1:0.00369615)1.000000:0.88964752)0.972000:0.2628

8596)0.936000:0.10789027)0.563000:0.04264862)0.470000:0.05994578)0.9
42000:0.26857196)1.000000:0.75267361,
((((((Phialocephala_scopiformis_XP_018071283.1:0.37369146,
((Pseudogymnoascus_sp._WSF_3629_OBT39068.1:0.01554800,
((Pseudogymnoascus_sp._VKM_F-4517__FW-2822__KFY90239.1:0.05699887,Ps
eudogymnoascus_sp._VKM_F-4517__FW-2822__KFY97971.1:0.01666826)0.8930
00:0.01280329,
((Pseudogymnoascus_sp._VKM_F-4520__FW-2644__KFZ23578.1:0.00528943,Ps
eudogymnoascus_sp._VKM_F-4518__FW-2643__KFY91446.1:0.01439595)0.9420
00:0.02032707,Pseudogymnoascus_sp._VKM_F-3775_KFY25049.1:0.11449644)
0.881000:0.01585606)0.914000:0.01797238)0.704000:0.00677835,
((Pseudogymnoascus_sp._VKM_F-4519__FW-2642__KFZ11099.1:0.00809815,Ps
eudogymnoascus_verrucosus_XP_018133051.1:0.00270092)0.000000:0.00000
010,Pseudogymnoascus_sp._24MN13_OBT53705.1:0.01082438)0.086000:0.016
39731)1.000000:0.41513283)0.936000:0.16571159,
(Paraphaeosphaeria_sporulosa_XP_018037498.1:0.39942906,
(Bipolaris_oryzae_ATCC_44560_XP_007692019.1:0.05348863,
(Bipolaris_maydis_ATCC_48331_XP_014074159.1:0.09328723,
(Bipolaris_victoriae_FI3_XP_014551814.1:0.00864007,Bipolaris_zeicola
_26-
R-13_XP_007718900.1:0.08253536)0.999000:0.08070351)0.893000:0.042502
13)0.998000:0.38170346)1.000000:0.71660362)0.821000:0.08038914,
(Trichoderma_virens_Gv29-8_XP_013952530.1:0.08841315,Trichoderma_har
zianum_KKO96736.1:0.05547093)1.000000:0.48762698)0.782000:0.07596771
,((Lepidopterella_palustris_CBS_459.81_OCK85208.1:0.22842455,
(Cenococcum_geophilum_1.58_OCL00839.1:0.07843668,Glonium_stellatum_O
CL03949.1:0.07317966)0.947000:0.07045522)1.000000:0.41356264,Aureoba
sidium_subglaciale_EXF-2481_XP_013340596.1:0.68840444)0.700000:0.107
17814)0.274000:0.06414738,
((Grosmannia_clavigera_kw1407_XP_014171803.1:0.43555987,Scedosporium
_apiospermum_XP_016646108.1:0.29870912)0.177000:0.07127164,
(Nectria_haematococca_mpVI_77-13-4_XP_003040478.1:0.23562563,
((Fusarium_avenaceum_KIL86360.1:0.07639573,
(Fusarium_graminearum_PH-1_XP_011323221.1:0.03246176,Fusarium_pseudo
graminearum_CS3096_XP_009259177.1:0.05241965)0.951000:0.04861019)0.9
91000:0.12037638,
((Fusarium_verticillioides_7600_XP_018747448.1:0.04000475,
(Fusarium_mangiferae_CVK91889.1:0.02189541,
((Fusarium_fujikuroi_KLO95416.1:0.00000025,Fusarium_fujikuroi_IMI_58
289_CCT67958.1:0.00601093)0.983000:0.02393500,
(Fusarium_proliferatum_ET1_CZR43844.1:0.00878194,Fusarium_proliferat
um_CVL12266.1:0.01182655)0.909000:0.01041959)0.049000:0.00520843)0.3
61000:0.01039445)0.451000:0.02653234,
(Fusarium_oxysporum_f._sp._pisi_HDV247_EXA31323.1:0.00652769,
(Fusarium_oxysporum_f._sp._cubense_tropical_race_4_54006_EXL91994.1:
0.01217760,(Fusarium_oxysporum_Fo47_EWZ36393.1:0.00605667,
((Fusarium_oxysporum_FOSC_3-a_EWY89805.1:0.00302789,
(Fusarium_oxysporum_f._sp._lycopersici_4287_XP_018239545.1:0.0030255
8,Fusarium_oxysporum_f._sp._vasinfectum_25433_EXM24697.1:0.00302250)
0.755000:0.00301220)0.801000:0.00303110,
(Fusarium_oxysporum_f._sp._lycopersici_MN25_EWZ80049.1:0.01541868,
(Fusarium_oxysporum_f._sp._raphani_54005_EXK79363.1:0.00904994,Fusar
ium_oxysporum_f._sp._radicis-
lycopersici_26381_EXL55858.1:0.00299062)0.791000:0.00303354)0.000000

:
0.00000017)0.000000:0.00000012)0.000000:0.00000016)0.820000:0.005560
04)0.788000:0.02920516)0.999000:0.18746947)0.997000:0.15212292)0.985
000:0.14226024)1.000000:0.40336395)0.216000:0.06480087,
(Rasamsonia_emersonii_CBS_393.64_XP_013328819.1:0.35010877,
((((Talaromyces_cellulolyticus_GAM39441.1:0.03892128,Talaromyces_ver
ruculosus_KUL85984.1:0.01756002)1.000000:0.33108413,
(Aspergillus_udagawae_GAO88321.1:0.09323082,
(Aspergillus_flavus_AF70_KOC16012.1:0.00320656,
(Aspergillus_oryzae_RIB40_BAE55371.1:0.00000001,Aspergillus_oryzae_R
IB40_XP_001817373.2:0.00000006)0.866000:0.00834896)0.999000:0.114769
76)1.000000:0.22629088)0.998000:0.19966335,
(Aspergillus_wentii_DTO_134E9_OJJ30262.1:0.39827761,
(Aspergillus_terreus_NIH2624_XP_001215889.1:0.34405792,
(Aspergillus_versicolor_CBS_583.65_OJJ07756.1:0.57095065,
((Penicillium_rubens_Wisconsin_54-1255_XP_002565677.1:0.07041096,
(Penicillium_expansum_XP_016595824.1:0.08156397,
(Penicillium_camemberti_CRL18857.1:0.04221399,Penicillium_griseofulv
um_KXG52483.1:0.07023032)0.891000:0.01934509)0.790000:0.01444015)0.9
97000:0.17520210,
(Penicillium_subrubescens_OKO95972.1:0.11603333,Penicillium_brasilia
num_CEJ62007.1:0.13534080)1.000000:0.26752836)0.979000:0.12823817)0.
627000:0.10569942)0.427000:0.04128406)0.428000:0.07683097)0.948000:0
.
07695984,Rasamsonia_emersonii_CBS_393.64_XP_013329590.1:0.49550543)0
.
526000:0.05533821)0.984000:0.24472710)1.000000:0.81634096)1.000000:0
.97920066)1.000000:0.54024752);
